# Dual pKa Lipid Nanoparticles for Lung‐tropic mRNA Delivery and pH‐Programmed Endosomal Escape

**DOI:** 10.1002/advs.77387

**Published:** 2026-09-09

**Authors:** Seong Gi Lim, Minju Lee, Yiming Wang, Eunjin Choi, Ji Sun Park, Kyungmin Park, Hayoung Jeon, Donghyun Kim, Donghyun Lee, Yeeun Lee, Kee‐Pyo Kim, Eunha Kim, Heebeom Koo

**Affiliations:** ^1^ Department of Medical Life Sciences and Department of Medical Sciences (Graduate School) College of Medicine The Catholic University of Korea Seoul Republic of Korea; ^2^ Department of Molecular Science and Technology Ajou University Suwon Republic of Korea; ^3^ Department of Chemical and Biomolecular Engineering University of Pennsylvania Philadelphia PA USA; ^4^ Southwest Research Institute San Antonio TX USA

**Keywords:** acute lung injury, dual pKa ionizable lipid, lipid nanoparticle, lung‐tropic

## Abstract

Lipid nanoparticles (LNPs) have enabled the clinical application of RNA therapeutics, including approved mRNA vaccines and siRNA medicines. However, their predominant accumulation in liver after systemic administration and inefficient endosomal escape remain key bottlenecks for productive cytosolic delivery and gene expression. Here, we introduce a synthetic ionizable lipid with a dual pKa property that is retained in formulated LNPs. We show that after intravenous injection, these dual pKa LNPs produce lung‐selective mRNA expression with higher potency than a cationic lipid‐rich comparator while remaining well tolerated. Using molecular dynamics simulations, we find that protonation state can support two distinct interaction modes between the ionizable lipid and endosomal membranes, suggesting a mechanistic basis for efficient endosomal escape. Moreover, the formulation maintains robust pulmonary expression and delivers therapeutic benefit in an acute lung inflammation model. These results establish a structure‐property relationship within the ionizable lipid‐based LNPs investigated here and identify dual apparent pKa behavior as a promising feature for potent and tolerable lung‐targeted RNA delivery.

## Graphical abstract

Lipid nanoparticles’ bottleneck lies in low endosomal escape efficiency and liver‐dominant delivery after intravenous administration. To overcome these limitations, we present a stepwise pH‐programmed lipid nanoparticle system enabled by our newly synthesized ionizable lipid. This system achieves robust mRNA expression exclusively in the lung and enhanced therapeutic activity in an acute lung injury model.

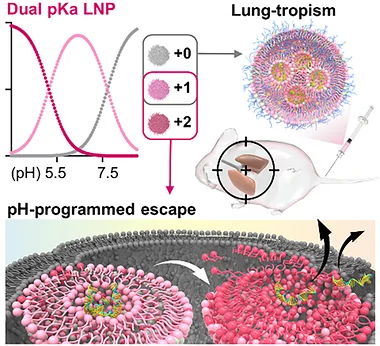

## 1 Introduction

Messenger RNA (mRNA)‐containing lipid nanoparticles (LNPs) have delivered major clinical impact through COVID‐19 vaccines [1, 2] and are expanding rapidly toward systemic gene therapy [3, 4]. Beyond intramuscular vaccination, recent efforts have increasingly focused on intravenous dosing for systemic applications [5, 6, 7], and conventional LNPs predominantly accumulate in the liver [8, 9]. To extend the therapeutic reach, extrahepatic targeting is increasingly pursued [5, 10], with particular interest in the lungs given the substantial unmet needs in acute injuries such as acute lung injury (ALI) or acute respiratory distress syndrome (ARDS) [11, 12, 13], where management remains largely supportive. However, this remains challenging because organ selectivity, cytosolic delivery, and tolerability are often influenced by overlapping formulation variables and therefore must be optimized simultaneously [7, 14, 15, 16, 17]. Selective organ targeting (SORT) strategies modulate organ tropism by incorporating charged lipids and have enabled lung tropism using formulations that pair an ionizable lipid with a permanently cationic lipid, such as DOTAP (1,2‐dioleoyl‐3‐trimethylammonium‐propane) [18, 19]. However, these permanently cationic components can exacerbate dose‐limiting cytotoxicity [20], particularly at therapeutically relevant doses, imposing a persistent trade‐off between delivery efficacy and safety.

**SCHEME 1:**
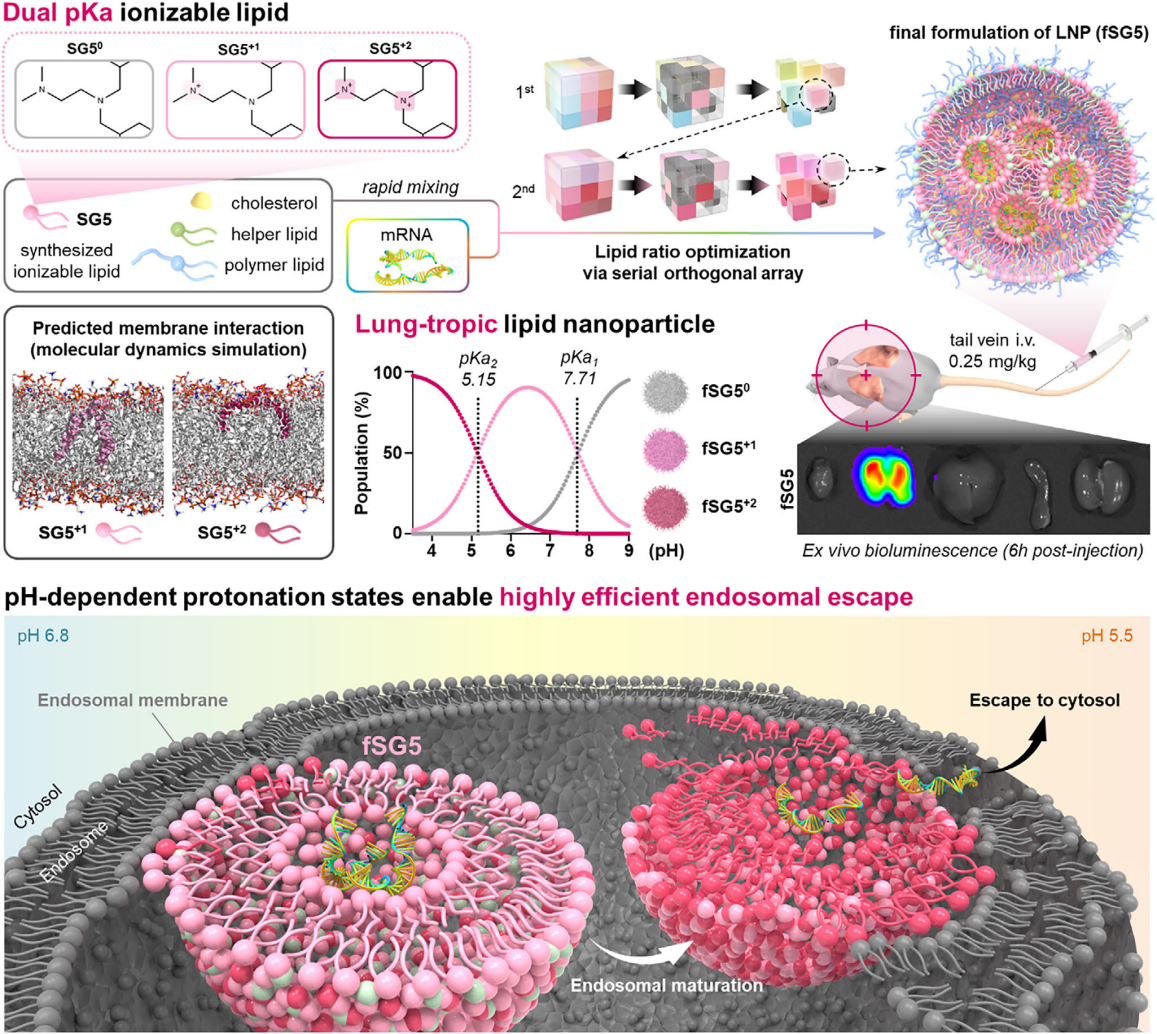
Schematic overview of dual pKa lipid nanoparticles for lung‐tropic mRNA delivery and pH‐programmed endosomal escape.

In parallel, inefficient endosomal escape remains a major bottleneck for mRNA delivery [21, 22], motivating the development of pH‐responsive designs that engage membranes along the endocytic route. Across cationic delivery systems including polymers [23] and membrane‐active peptides [24], endo‐lysosomal acidification is frequently leveraged to increase charge accessibility and strengthen interactions with anionic membranes. We therefore hypothesized that LNPs exhibiting two discrete apparent pKa transitions can access distinct charge states across the endocytic pH range, widening the window for productive membrane interactions and cytosolic release. Whether dual‐transition behavior provides practical advantages over conventional ionizable lipids without compromising tolerability remains unclear. Here, we first established that SG5, a lead candidate from our ionizable lipid series, forms LNPs that exhibit two discrete apparent pKa transitions (Scheme 1). We then examined whether this dual transition behavior could “program” charge accessibility across the trafficking pH gradient within a single‐molecule design.

We report a dual transition ionizable lipid (SG5) and a final, lipid ratio‐optimized SG5‐based LNP formulation (fSG5), identified through helper lipid screening and two rounds of orthogonal‐array optimization. We benchmarked fSG5 against a reported lung‐tropic SORT comparator based on C12‐200 across in vitro and in vivo expression models, including luciferase and ROSA26‐Cre reporters. To connect the pH‐dependent behavior to endosomal escape, we evaluated membrane engagement and escape using Laurdan assay, hemolysis assay, and confocal imaging. Molecular dynamics simulations suggest a mechanistic basis for the pH‐responsive engagement of ionizable lipids with endosomal membranes by revealing protonation state‐dependent interaction modes. We then profiled the tolerability in vitro and after a single high intravenous dose in healthy mice. Finally, we tested the therapeutic relevance by delivering Nrf2 (nuclear factor erythroid 2‐related factor 2) mRNA in a lipopolysaccharide (LPS)‐induced ALI model and assessing inflammation, edema and Nrf2 pathway engagement.

## 2 Results and Discussion

### 2.1 Synthesis of SG‐Series Ionizable Lipids and Helper Lipid Selection

**FIGURE 1:**
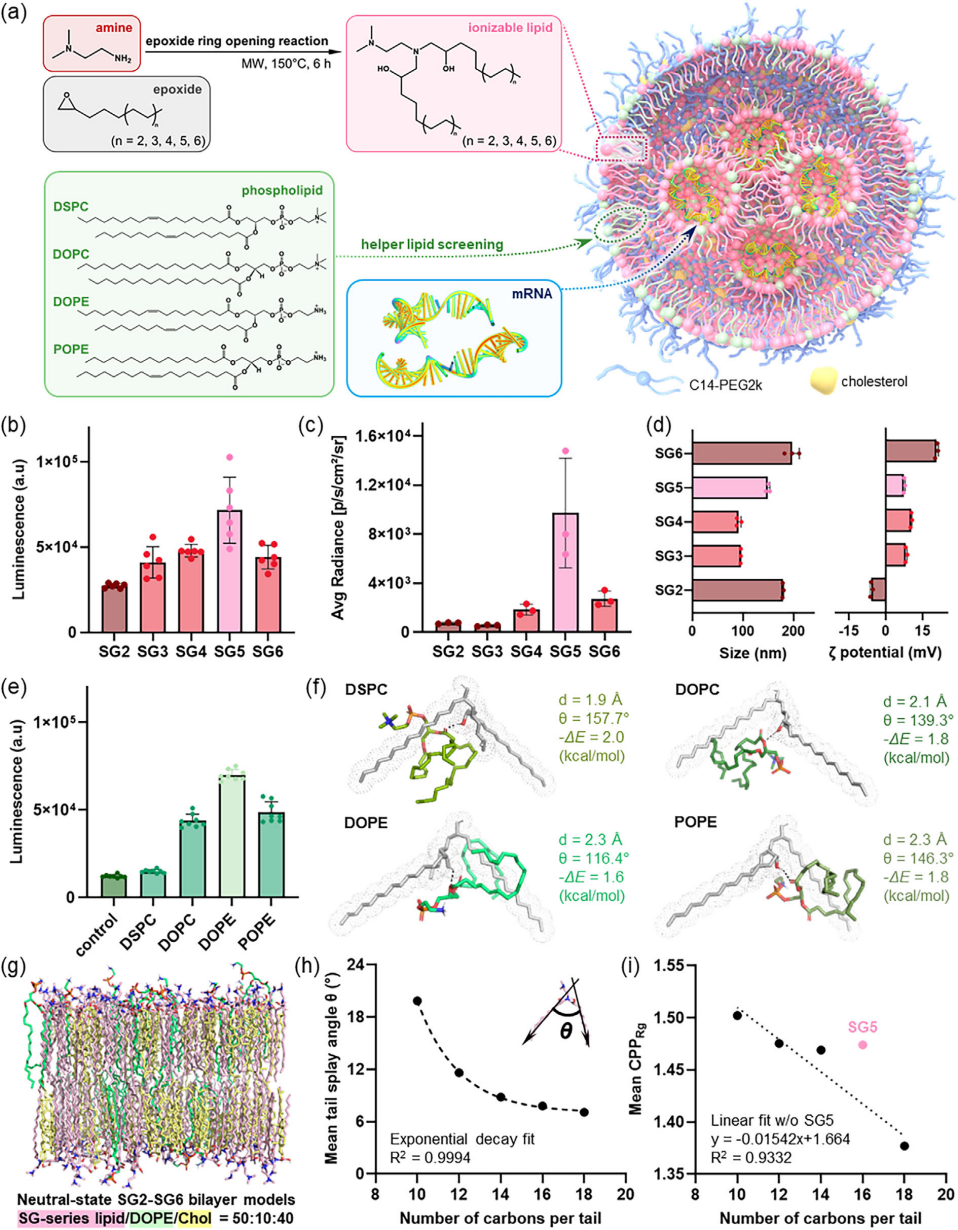
Synthesis of SG‐series ionizable lipids and initial formulation screening. (a) Schematic illustration of the synthesis of SG‐series ionizable lipids and the composition of the SG‐series LNPs. (b) In vitro luciferase expression in HeLa cells treated overnight with SG‐series LNPs. (c) Average radiance flux of Luc expression in the lungs of CD‐1 mice treated with SG‐series LNPs (*n = 3* biological replicates). (d) Hydrodynamic diameter and zeta potential of SG‐series LNPs measured by DLS. (e) Molecular docking simulation between the selected ionizable lipid (SG5) and helper lipids. LNPs were administered at 50 ng mRNA per well (96‐well plate) for in vitro assays and 0.25 mg mRNA/kg for in vivo studies. (g) Molecular dynamics simulations of bilayer systems containing neutral‐state SG2, SG3, SG4, SG5, or SG6 ionizable lipids at the indicated molar composition. (h) Mean tail splay angle of SG‐series ionizable lipids as a function of the number of carbon atoms per lipid tail. The inset schematically illustrates the angle (*θ*) between the representative vectors of the two hydrocarbon tails. (i) Time‐averaged CPP_Rg_ values of SG‐series ionizable lipids with a linear regression fit to SG2, SG3, SG4, and SG6.

We synthesized ionizable lipids via epoxide ring‐opening with N,N‐dimethylethylenediamine (DMEDA), as shown in Figure 1a. Using five commercially available epoxides, we generated an ionizable lipid series, SG2‐SG6, defined by the carbon tail length. Their chemical structures were evaluated by NMR analysis (Figures S1–S5). The in vitro (Figure 1b) and in vivo (Figures 1c and S8) efficacy of the SG‐series was evaluated in HeLa cells and lungs of CD‐1 mice, respectively, using a luciferase assay with LNPs using the conventional formulation (CVN), consisting of the ionizable lipid:helper lipid:cholesterol:PEG‐lipid molar ratio of 50:10:38.5:1.5 [25], and a total lipid:mRNA weight ratio of 40:1 [19]. Particle size and zeta potential were also measured (Figure 1d). Except for SG2 (10‐carbon tail) and SG6 (18‐carbon tail), all LNP diameters were less than 150 nm, indicating that they were sufficiently small for efficient cellular uptake [26, 27]. Zeta potential analysis showed that most formulations carried positive surface charges (SG3, +8.29 mV; SG4, +10.4 mV; SG5, +7.56 mV; and SG6, +20.8mV), whereas SG2 exhibited a negative charge (−5.81 mV). Based on these results, SG3, SG4, and SG5 were considered promising candidates for further evaluation. Among these, SG5 showed the highest efficacy in both in vitro and in vivo systems, and we therefore selected SG5 for subsequent studies.

Optimization of SG5‐based LNPs began with identification of the most suitable helper lipid. Four widely used phospholipids were selected as helper lipid candidates: DSPC, DOPC, DOPE, and POPE [28, 29]. In vitro luciferase assays indicated that DOPE exhibited the highest efficacy (Figure 1e). When interactions between SG5 and helper lipids were analyzed by molecular docking (Figure 1f), DSPC showed the shortest hydrogen bond distance (1.9 Å) and the largest angle (157.7°), consistent with a strong hydrogen bond geometry. Interactions with DOPC and POPE were characterized by distances and angles of 2.1 Å/139.9° and 2.3 Å/146.3°, respectively. While other helper lipids exhibited hydrogen bond‐like interactions, DOPE formed an interaction of 2.3 Å and 116.4°, which did not fall within the conventional hydrogen bond range [30]. Thus, the interaction between DOPE and SG5 is more appropriately categorized as a weaker polar association (e.g., dipole‐dipole interaction), and this reduced association may contribute to the enhanced endosomal escape and superior efficacy observed with DOPE [31]. Notably, these docking trends were concordant with the in vitro luciferase ranking, both identifying DOPE as the optimal helper lipid for the SG5‐based LNPs.

To examine whether the superior efficacy of SG5 was associated with its molecular organization within LNPs, we conducted molecular dynamics simulations using LNP‐mimicking bilayer models. Following a previously reported bilayer modeling approach, PEG‐lipid was omitted [32, 33], and each SG‐series lipids, DOPE, and cholesterol were modeled at a molar ratio of 50:10:40 (Figure 1g). Over the final 500 ns of the simulations, we first examined the tail‐length‐dependent behavior of the ionizable lipids. The mean tail splay angle [34] followed an exponential decay trend with increasing carbon number per tail (Figure 1h). Similarly, both the local Voronoi area [35] of SG‐series lipids and the global area per lipid (APL) of the membrane progressively decreased with increasing tail length (Figure S9). These behaviors were consistent with previous observations that increasing saturated acyl chain length can reduce the average molecular area in homologous lipid bilayers [36, 37]. In contrast, analysis of the radius‐of‐gyration‐based critical packing parameter (CPP_Rg_) [32] revealed a distinct behavior of SG5. The mean CPP_Rg_ values of SG2, SG3, SG4, and SG6 followed an empirical linear trend (R^2^ = 0.9332), whereas SG5 exhibited a positive deviation and reduced the R^2^ to 0.6897 when included in the fit (Figures 1i and S10). These results suggest that the distinctive behavior of SG5 was not associated with abnormal tail splay or lateral membrane packing, but rather with the relative spatial distribution of its headgroup and tail regions. Given the previously reported positive association between CPP_Rg_ and gene expression efficacy [32], the elevated CPP_Rg_ of SG5 may contribute to its enhanced efficacy.

### 2.2 Optimization of the Selected SG5 Ionizable Lipid‐Based LNPs

**FIGURE 2:**
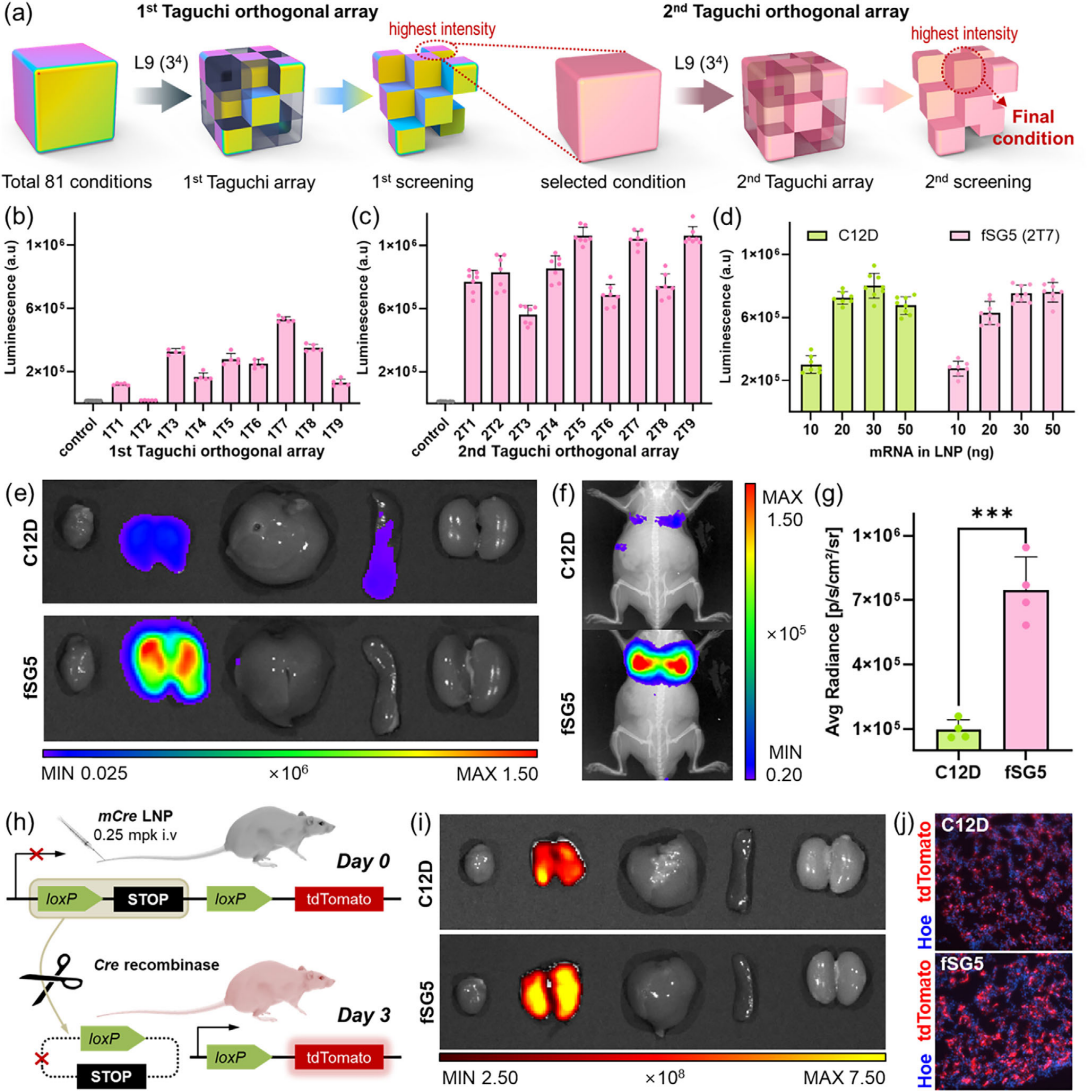
Systemic screening of LNP formulation variables using the Taguchi design. (a) Overview of the Taguchi screening strategy. Luciferase expression measured in a 96‐well assay for (b) the first and (c) the second Taguchi screening. (d) Comparison of dose‐dependent transfection efficiency between fSG5 and C12D LNPs. (e) Ex vivo luminescence images of major organs from CD‐1 mice intravenously injected with lung‐targeting LNPs. (f) Whole‐body bioluminescence images of the same mice. (g) Quantification of average radiance flux in ex vivo lungs (*n = 4* biological replicates). (h) Schematic of Cre recombinase‐mediated tdTomato expression in ROSA26 reporter mice. (i) Ex vivo fluorescence images of major organs from ROSA26 mice at 3 days post intravenous mRNA injection. (j) Representative fluorescence images of lung cryosections with Hoechst 33342 nuclear staining. Scale bar, 100 µm. LNPs were administered at 50 ng mRNA per well (96‐well plate) for in vitro assays and 0.25 mg mRNA/kg for in vivo studies. Unpaired, two‐tailed Student's t‐test, *p < 0.05, **p < 0.01, ***p < 0.0001, ****p < 0.00001.

Given the importance of lipid composition in LNP efficacy, we used two sequential Taguchi orthogonal arrays to identify the optimized ratio [38]. Instead of testing all 81 conditions in a full factorial, each array comprised only nine representative experiments (Figure 2a). Before defining the factor levels, we referred to several well‐established LNP formulations [39, 40, 41, 42, 43]. Preliminary data indicated higher efficacy at higher ionizable lipid fractions, and the initial conditions were defined according to the two top‐performing compositions (Figures S11 and S12). The complete lipid ratios are presented in Table S1. In the first array, the ratio 1T7 showed the highest efficacy, so the second array was subsequently designed around 1T7 (Figure 2b). In the second array, the average luciferase expression was increased overall relative to the first, with 2T5, 2T7, and 2T9 showing the highest in vitro efficacy (Figure 2c). To select the final composition, we evaluated these three ratios in vivo by whole‐body luminescence (Figure S13), and 2T7 yielded the highest signal. Therefore, SG5‐based lipid nanoparticles at the 2T7 ratio were established as the final optimized LNP formulation (fSG5).

Most ionizable lipids to date (e.g., C12‐200 and cKK‐E12) carry 12 to 14‐carbon tails and favor hepatic uptake [44], which hinders pulmonary delivery. In contrast, SG5, which has 16‐carbon tails, directed LNPs to the lungs across a wide range of molar ratios (Figures S8 and S11). This pattern aligns with recent reports that, within a lung‐targeting ionizable lipid series, increasing tail length correlates with higher lung mRNA expression [45]. Accordingly, we compared fSG5 with a well‐known and efficient lung‐targeted SORT system, C12‐200‐based LNPs containing 50% DOTAP (C12D) [18]. C12D and fSG5 produced comparable luciferase expression in vitro (Figure 2d). In vivo, however, fSG5 was notably more potent than C12D (Figure 2e–g). This advantage was maintained when fSG5 and C12D were compared at the same total lipid:mRNA weight ratio of 40:1 (Figure S14). To cross‐validate these findings and assess the gene‐editing potential, we used ROSA26 reporter mice that received Cre recombinase mRNA (Figure 2h). When the inserted Cre mRNA is translated, the biosynthesized Cre recombinase enzyme recognizes the two loxP sites and removes the inserted STOP signal. When the inhibition signal is eradicated, tdTomato protein is expressed and therefore signals can be collected [46, 47]. As a result, fSG5 yielded higher tdTomato expression, and the sliced tissue images after cryosection further confirmed its superior efficacy (Figure 2i,j).

### 2.3 Dual Transition Apparent pKa Behavior of fSG5 and Analogue Analysis

**FIGURE 3:**
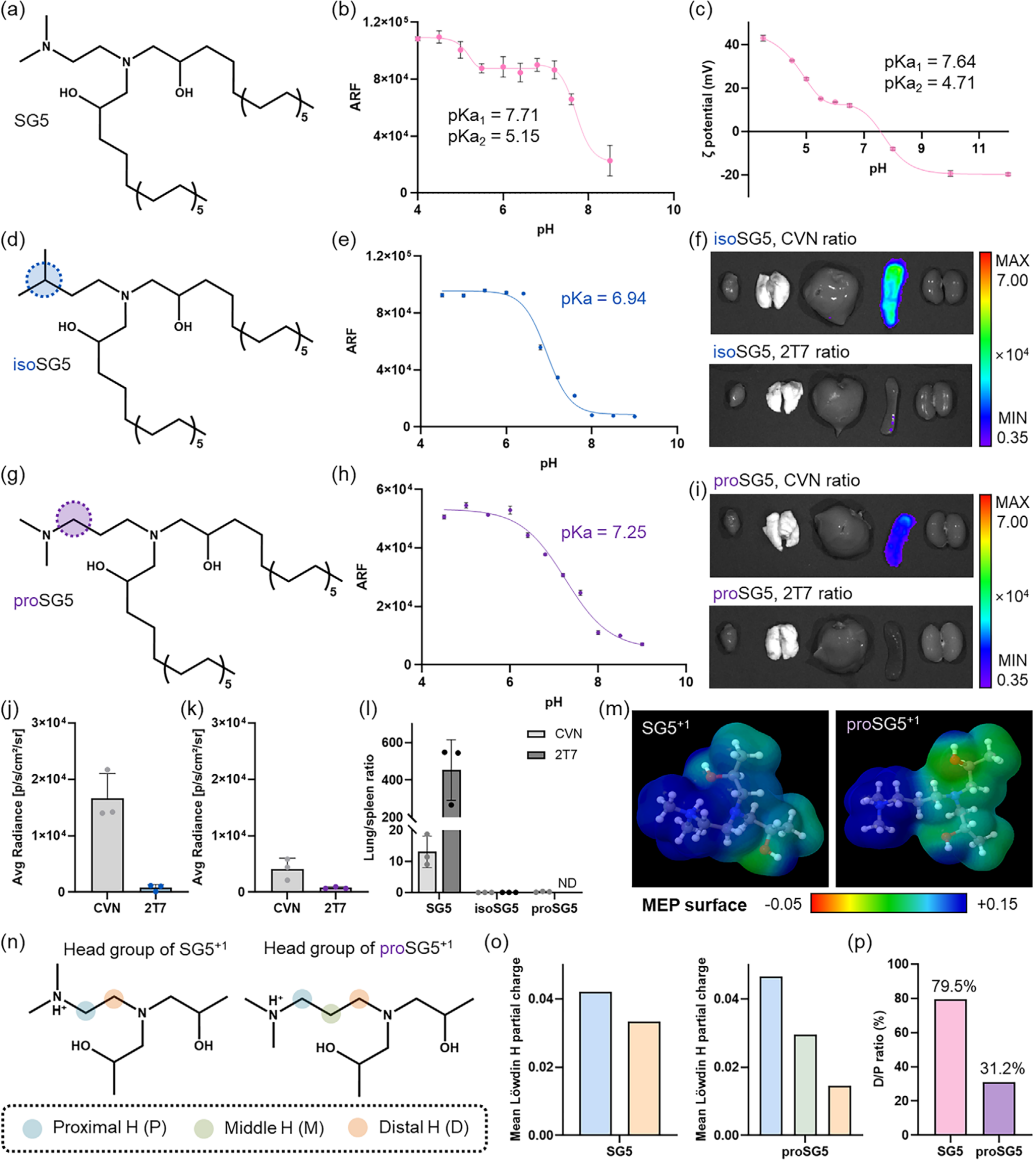
Dual apparent pKa transitions of fSG5 and analysis of ionizable lipid analogues. (a) Chemical structure of SG5. Apparent pKa determination of fSG5 by (b) TNS fluorescence over pH 4.0–8.5 and (c) zeta potential titration over pH 3–12. Chemical structures of (d) isoSG5 and (g) proSG5. TNS fluorescence profiles of (e) isoSG5‐ and (h) proSG5‐based LNPs formulated at the CVN ratio over pH 4.5–9.0 for apparent pKa determination. Ex vivo IVIS images of organs from mice treated with (f) isoSG5‐ and (i) proSG5‐based LNPs formulated at the indicated lipid ratios. Quantification of splenic average radiance following treatment with (j) isoSG5‐ and (k) proSG5‐based LNPs. (l) Lung‐to‐spleen radiance ratios of SG5‐ and analogues‐based LNPs. (m) Molecular electrostatic potential surfaces of SG5^+1^ and proSG5^+1^. (n) Head group structures of mono‐protonated SG5 and proSG5 with schematic assignment of the proximal, middle, and distal H positions. (o) Mean Löwdin partial charges of the H atoms in the corresponding regions of SG5 and proSG5. (p) Distal‐to‐proximal ratios of the mean Löwdin H partial charges of SG5 and proSG5.

Next, we investigated the basis underlying the superior performance of fSG5 over C12D. Because fSG5 and C12D exhibited similar particle sizes and polydispersity index (PDI) while C12D showed a more positive zeta potential (Figure S15), we next investigated physicochemical differences originating from the ionizable lipid structure. The intrinsic pKa values of the SG5 lipid were first determined by Sirius T3 titration [48]. Across pH 2–11, two protonation events were identified, with pKa values of 7.34 ± 0.02 and 2.50 ± 0.00 (Figure S16). This result suggests two separable protonation transitions and motivates measurements at the LNP level. We therefore measured the apparent pKa behavior of fSG5 using two complementary assays: TNS fluorescence and ζ‐potential titration [49]. Both approaches consistently revealed two distinct pKa values in fSG5 (Figure 3b,c). Zeta potential titration yielded values of 7.64 and 4.71, whereas the TNS assay yielded values of 7.71 and 5.15. The small discrepancy between the two methods (<0.5) was within the expected inter‐assay variability [49]. Because the TNS assay is more widely accepted for determining the apparent pKa of LNPs [50], the TNS values were adopted as the representative pKa values of fSG5. Despite ionizable lipids containing multiple amines, LNP formulations often exhibit a single dominant apparent transition within physiologically relevant pH windows [51, 52], which is also shown in the case of C12D in Figure S17. In this context, the clear two‐transition behavior observed for fSG5 is notable and supports our mechanistic analyses.

To examine whether this behavior depended on the number or spacing of the amines, we synthesized two SG5 analogues. A single‐amine analogue, designated as isoSG5, was prepared by replacing single amine containing moiety with a carbon containing group (Figure 3d). A single apparent pKa of 6.94 was exhibited when isoSG5‐based LNPs were formulated at the CVN ratio (Figure 3e). In vivo luciferase imaging showed predominant splenic expression at both the CVN and 2T7 ratios, with minimal lung expression (Figure 3f). We next synthesized proSG5, in which the two‐carbon spacer between the amines of SG5 was extended to a three‐carbon spacer (Figure 3g). Interestingly, a single apparent pKa value of 7.25 was shown in proSG5‐based LNPs at CVN ratio (Figure 3h). A weak luciferase expression predominantly in the spleen was shown in proSG5‐based LNPs in CVN ratio, and no signal was observed at 2T7 ratios (Figure 3i). The splenic luciferase signals from LNPs based on isoSG5 and proSG5 were quantified in Figure 3j,k, respectively. The relatively high apparent pKa values and spleen‐dominant distribution of the analogue‐based LNPs were consistent with a recent report of a spleen‐tropic LNP exhibiting a similarly high apparent pKa [53]. To compare the lung‐targeting property of SG5‐based LNPs, we compared the lung‐to‐spleen ratio of each ionizable lipids in both CVN or 2T7 ratio (Figure 3l). The comparison between SG5 and isoSG5 revealed that the removal of one amine shifted the organ distribution from the lungs to the spleen. Notably, increasing the distance between the two amines in proSG5 abolished both lung selectivity and high transfection efficacy. To investigate this dependency on spatial arrangement of two amines, we compared the molecular electrostatic potential (MEP) distributions of mono‐protonated SG5 (SG5^+1^) and proSG5 (proSG5^+1^). The electrostatic influence of the protonated amine extended toward the second, non‐protonated amine in SG5^+1^, whereas the positive potential of proSG5^+1^ was more localized around the protonated site (Figure 3m). This suggested interaction between the closely spaced amines of SG5. The mean partial charges of hydrogens located proximal, middle, or distal to the protonated amine were further evaluated (Figure 3n) using Löwdin (Figure 3o) and Mulliken population analyses (Figure S18). In SG5^+1^, the mean Löwdin charges of the proximal and distal hydrogens were +0.04207 and +0.03346, respectively. In proSG5^+1^, the proximal, middle, and distal values were +0.04648, +0.02963, and +0.01450, respectively. Accordingly, the distal‐to‐proximal charge retention ratio was 0.795 for SG5^+1^ and 0.312 for proSG5^+1^ (Figure 3p). Mulliken analysis showed the same trend, with retention ratios of 0.822 and 0.354, respectively (Figure S19). Thus, the electrostatic perturbation induced by protonation was propagated across the SG5 head group but was markedly attenuated by the longer spacer in proSG5. Collectively, these results suggest that the closely spaced amines of SG5 are electronically interdependent. Following the first protonation event, electrostatic perturbation of the intervening head group region may alter the environment of the second amine, making its subsequent protonation less favorable, thereby contributing to the widely separated intrinsic pKa values and dual apparent pKa behavior of fSG5. In contrast, increasing the distance between the amines attenuated this coupling, allowing the two protonation events to occur more independently and resulting in a single dominant apparent transition, as is commonly observed for other multiamine ionizable lipids. Together, these findings support a structure‐property relationship among the precise amine arrangement of SG5, its dual‐transition behavior, and the lung‐selective expression of SG5‐based LNPs.

### 2.4 pH‐Dependent Membrane Properties and Endosomal Escape of fSG5

**FIGURE 4:**
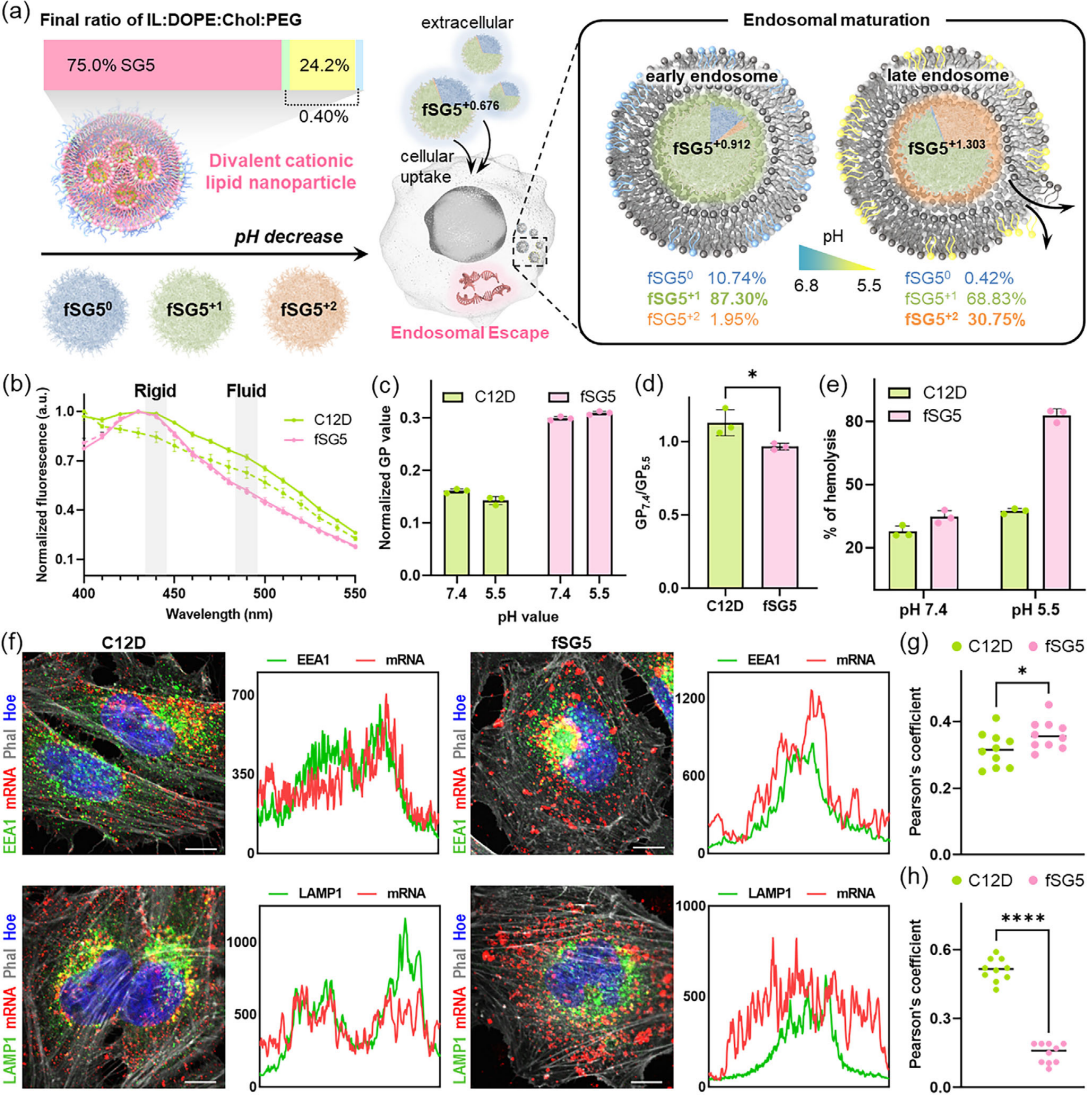
pH‐Dependent membrane behavior and endosomal trafficking of LNPs. (a) Schematic illustration of the fSG5 LNP composition and inferred charge distribution based on apparent pKa. (b) Emission spectra (400–550 nm) of Laurdan dyes mixed with LNPs at a 1:250 ratio. (c) Normalized generalized polarization (GP) values at pH 7.4 and pH 5.5. (d) GP ratio (pH 7.4/pH 5.5). (e) Hemolysis measured at pH 7.4 and pH 5.5. (f) Confocal imaging of colocalization between Cy5‐labeled mRNA delivered by LNPs and endosomal compartments along the endo‐lysosomal pathway. Pearson's correlation coefficients for C12D and fSG5 with (g) EEA1 and (h) LAMP1. For confocal microscopy, LNPs were administered at 500 ng mRNA per well (12‐well plate). Scale bar, 10 µm. Unpaired, two‐tailed Student's t‐test, *p < 0.05, **p < 0.01, ***p < 0.0001, ****p < 0.00001.

Based on these findings, we propose that fSG5 exists in three charge states across the delivery pathway: neutral, monovalent, and divalent cationic (Figure 4a). In the bloodstream, a slight positive charge may promote cellular uptake and lung targeting [20]. In early endosomes, the majority of fSG5 transitions to a monovalent cationic state, which initiates membrane destabilization. As the pH decreases during endosomal maturation, the fraction of divalent cationic species increases, amplifying the destabilization [54]. Together, these results support a model in which dual transition behavior enables stepwise charge‐state switching along the endocytic pathway, broadening charge accessibility during maturation, and thereby facilitating mRNA cargo release.

Since smaller differences in LNP rigidity between physiological pH (7.4) and endosomal pH (5.5) are associated with improved delivery efficacy [55], we profiled C12D and fSG5 with Laurdan (Figure 4b). Laurdan emission reports interfacial packing or hydration: more ordered (rigid) membranes show higher intensity near 440 nm, whereas less ordered (more hydrated) membranes peak near 490 nm [56, 57]. Normalized generalized polarization (GP) values indicated that C12D was more disordered than fSG5 at both pH conditions (Figure 4c). Importantly, only fSG5 exhibited an increase in GP at pH 5.5, resulting in a smaller GP ratio (7.4/5.5) of fSG5 (Figure 4d). This behavior is consistent with prior Laurdan GP analyses, which showed that maintaining interfacial packing under acidic conditions is associated with higher protein expression [55]. We then functionally evaluated endosomal escape by performing a pH‐dependent hemolysis assay using red blood cells as a membrane‐rupture surrogate [58]. At pH 7.4, both LNPs induced modest hemolysis (27.7% and 34.7%, respectively). At pH 5.5, hemolysis by fSG5 increased sharply to 83.1%, compared to 37.5% for C12D (Figure 4e). These results support the conclusion that fSG5 has a greater endosomal escape capacity than C12D. Finally, endosomal escape was directly examined using confocal microscopy with immunostaining for EEA1 (early endosome marker) and LAMP1 (lysosome marker) (Figure 4f) [59]. Colocalization with EEA1 was comparable between C12D and fSG5, with Pearson's coefficients of 0.32 and 0.36, respectively (Figure 4g). In contrast, lysosomal colocalization differed markedly, with C12D showing moderate overlap (0.51) and fSG5 showing very low overlap (0.15) (Figure 4h). The reduced lysosomal colocalization of fSG5 supports more efficient escape of its mRNA payload during endosomal maturation, thereby reducing lysosomal degradation [60].

### 2.5 Molecular Dynamics Analysis of Protonation‐Dependent Membrane Engagement

**FIGURE 5:**
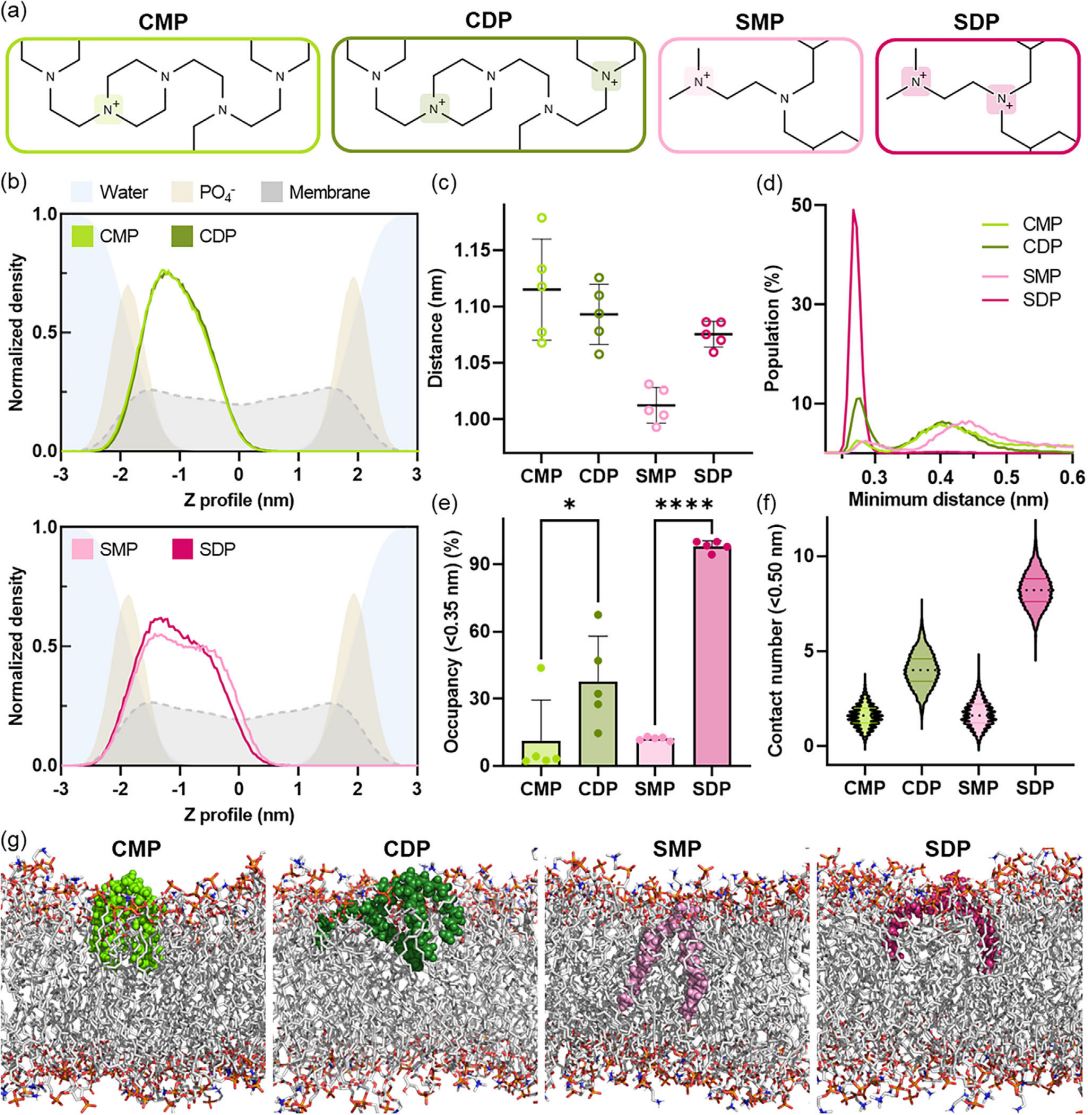
Molecular dynamic simulations under an endosome‐mimicking environment. (a) Naming convention for protonation states: C12‐200 (CMP, mono‐protonated; CDP, di‐protonated) and SG5 (SMP, mono‐protonated; SDP, di‐protonated). (b) System overview illustrated by z‐density profiles of the C12‐200 and SG5 systems. (c) Center‐of‐mass distance between each ionizable lipid and the membrane. (d) Minimum distance between protonated amines of ionizable lipids and phosphate oxygen atoms of the membrane lipids (BMP/DOPE/DOPC). (e) Contact occupancy within a 0.35 nm cutoff. (f) Contact number within a 0.50 nm cutoff. (g) Representative snapshots of each ionizable lipid system visualized in PyMOL. Color scheme: SMP (pink), SDP (hot pink), CMP (lime), CDP (forest green). The lipid bilayer is colored by element: carbon (gray), hydrogen (light gray), nitrogen (blue), oxygen (red), and phosphorus in phosphate groups (orange). One‐way ANOVA with Tukey's multiple‐comparisons test, *p < 0.05, **p < 0.01, ***p < 0.0001, ****p < 0.00001.

We performed molecular dynamics (MD) simulations under endosome‐mimicking conditions to probe membrane engagement relevant to endosomal escape, focusing on the distinct behaviors of C12‐200 and our synthesized ionizable lipid, SG5. Based on the Sirius T3 titration, C12‐200 also exhibited two apparent pKa values of 5.35 and 6.76 (Figure S20). We therefore compared the mono‐ and di‐protonated states of each ionizable lipid, with protonation sites and nomenclature summarized in Figure 5a. For C12‐200, protonation sites were assigned to the protonatable amines based on the piperazine moiety and the local steric environment imposed by the hydrophobic tails [61, 62].

An overview of the simulated systems is presented in Figure 5b. To assess how each component partitions relative to the bilayer along the membrane normal, we computed the number density profiles of the LNP components and membrane lipids along the z axis of the simulation box. C12‐200 displayed similar density profiles across the protonation states, whereas SG5 showed pronounced state dependence. Both SMP (+1, mono‐protonated) and SDP (+2, di‐protonated) were more bilayer‐center proximal than CMP (+1, mono‐protonated) or CDP (+2, di‐protonated) were. Within SG5, however, SMP partitioned closer to the bilayer center, whereas SDP remained closer to the interface. This ordering was consistent with the vertical distance between the lipid center of mass and the bilayer center of mass (Figure 5c). We used this center‐of‐mass distance as a bilayer penetration metric, following prior atomistic MD analyses of ionizable lipid insertion [63]. In our simulations, increased protonation of C12‐200 tended to correlate with a shorter distance to the bilayer center. In contrast, SG5 showed the opposite trend, with SMP exhibiting a shorter distance to the bilayer center than SDP, suggesting that SDP adopts a distinct interaction mode rather than simply increasing the bilayer penetration with increasing charge.

As the membrane model is enriched in bis(monoacylglycerol)phosphate (BMP), an anionic endosomal lipid, we hypothesized that electrostatic interactions between protonated amines of ionizable lipids and phosphate groups of phospholipids could compete with or redirect bilayer penetration. Accordingly, we quantified phosphate proximity as the minimum distance between phosphate oxygen atoms and protonated amine nitrogen atoms (Figure 5d). SDP exhibited a single sharp peak near 0.3 nm, consistent with a tight phosphate‐proximal association, whereas other conditions showed additional populations beyond approximately 0.35 nm. We next quantified the fraction of frames in close contact using a 0.35 nm cutoff (Figure 5e). SDP showed a high occupancy of phosphate‐proximal contacts at 98.07%, whereas SMP showed a much lower occupancy at 12.17%. C12‐200 showed the same directionality, with phosphate‐proximal occupancy increasing with protonation, from 11.18% for CMP to 37.75% for CDP. To capture the extent of the interaction beyond a single minimum distance, we further analyzed the number of contacts between protonated amine nitrogen and phosphate oxygen within 0.5 nm (Figure 5f). SDP exhibited markedly higher contact numbers than the other conditions, supporting a multipoint and persistent phosphate‐proximal anchoring mode rather than transient proximity.

Together, these analyses support two distinct membrane engagement modes for SG5 under endosome‐like conditions. SMP preferentially exhibits greater bilayer penetration, whereas SDP favors a phosphate‐proximal anchoring mode at the membrane interface, likely promoted by electrostatics in BMP‐rich membranes. Phosphate‐proximal anchoring has been discussed as an early step in certain membrane‐interaction pathways, including those of cell‐penetrating peptides [64]. Our results suggest that SDP may leverage a related principle for productive membrane engagement under endosomal conditions. We speculate that access to multiple protonation‐dependent engagement modes may contribute to the high potency of the SG5‐based LNPs by supporting productive membrane interactions across the endosomal pH range.

### 2.6 In Vitro Cytotoxicity and In Vivo Tolerability of fSG5

**FIGURE 6:**
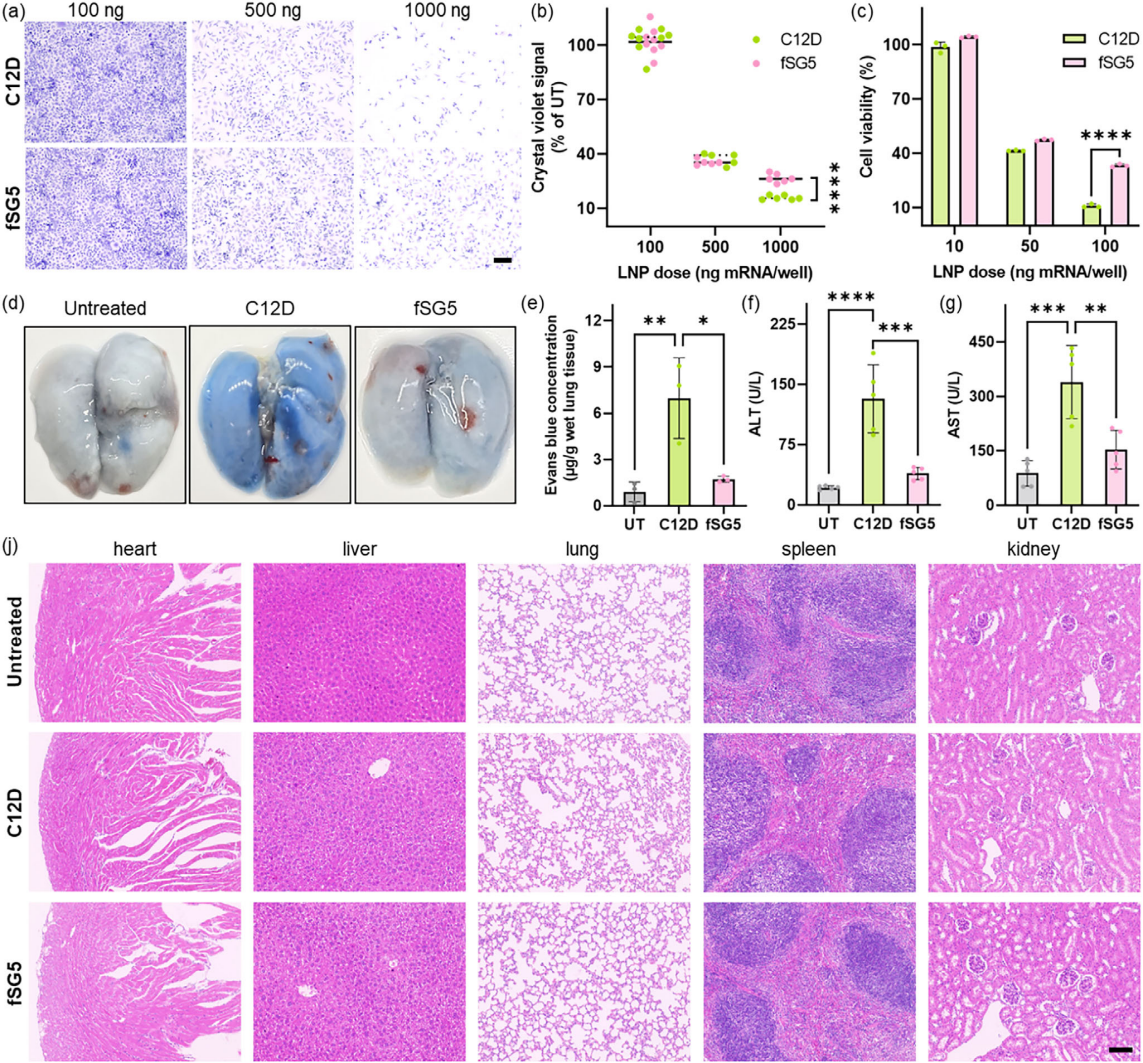
Biocompatibility assessment of lung‐tropic LNPs. (a) Crystal violet staining of HeLa cells in 12‐well plates after overnight treatment with lung‐tropic LNPs at the indicated LNP doses (100, 500, and 1000 ng mRNA per well). Scale bar, 200 µm. (b) Quantification of crystal violet staining by measuring absorbance at 575 nm; after solubilization, samples were transferred to a 96‐well plate for absorbance reading. (c) CCK‐8 assay showing dose‐dependent cytotoxicity in 96‐well plates. (d) Representative lung images and (e) quantification of Evans Blue dye extravasation as an indicator of vascular permeability (*n = 3* biological replicates). (f,g) Blood chemistry profiles of CD‐1 mice at 24 h after high‐dose intravenous administration of LNPs: (f) ALT (g) AST (*n = 5* biological replicates). (h) Representative H&E stained sections of major organs harvested from the same mice. Scale bar, 100 µm. UT, untreated. In vitro LNP doses are reported as ng mRNA per well as indicated. For in vivo toxicity tests, mice received 1.0 mg mRNA/kg intravenously. Unpaired, two‐tailed Student's t‐test (b, c) or one‐way ANOVA with Tukey's multiple‐comparisons test (e–g), *p < 0.05, **p < 0.01, ***p < 0.0001, ****p < 0.00001.

Ionizable lipids often present a toxicity‐efficacy trade‐off [65], and since fSG5 uses a relatively high molar fraction of ionizable lipids, we profiled the tolerability of fSG5 in vitro and in vivo. Crystal violet staining revealed a dose‐dependent reduction in viability for both LNPs, with fSG5 consistently preserving higher viability than C12D at the highest tested dose (Figure 6a,b). This pattern was corroborated by a complementary CCK‐8 assay, in which fSG5 exhibited significantly lower cytotoxicity than C12D at the highest dose (Figure 6c). Collectively, these assays indicated that fSG5 confers improved cellular tolerability at matched doses while maintaining its delivery performance.

Next, we evaluated the tolerability in healthy mice using a single high intravenous dose (1.0 mg/kg mRNA dose). In the Evans blue (Miles) assay [66], lungs from C12D‐treated mice showed greater dye extravasation than those from fSG5‐treated mice, whereas fSG5 remained similar to the untreated control (UT) under these conditions (Figure 6d). Quantification of dye content in lung tissue (µg per g of wet lung) supported this difference, with pulmonary leakage of fSG5 remaining similar to that of UT and lower than that of C12D at the same dose (Figure 6e). Serum chemistry measured 24 h after dosing further corroborated these findings [67]. ALT and AST levels did not differ between fSG5 and UT, whereas C12D showed higher values (Figure 6f,g). ALT levels remained within the reference interval for CD‐1 mice [68]. The mild, isolated AST elevation with C12D may reflect a transient, non‐hepatocellular response from extrahepatic sources [69, 70].

Histopathological examination of the heart, lung, liver, spleen, and kidney revealed no major lesions in any of the groups. Lung sections from LNP‐treated mice were unremarkable, with no overt structural damage, consistent with the original SORT study reporting no apparent tissue injury after treatment in C57BL/6 mice. This discordance between functional readouts (vascular permeability and serum chemistry) and histology suggest that C12D increases vascular permeability and systemic stress before overt histological lesions become detectable. Collectively, these results indicate the improved tolerability of fSG5 relative to the comparator at the tested high dose.

To further evaluate the inflammatory responses following intravenous administration of fSG5, mice received either a single or repeated injection of fSG5 at an mRNA dose of 0.30 mg/kg (Figure S21). Serum TNF‐α and IL‐6 levels measured at 6 and 24 h after the final administration showed no marked elevation relative to those in untreated control mice. Pulmonary expression of myeloperoxidase (MPO) and inducible nitric oxide synthase (iNOS) was also assessed in the same animals, with no marked increases detected by Western blot analysis. Together, these results indicate that repeated administration of fSG5 was not associated with marked systemic cytokine elevation or pulmonary induction of the inflammatory markers examined.

### 2.7 Therapeutic Efficacy of fSG5 in LPS‐Induced Acute Lung Injury Model

**FIGURE 7:**
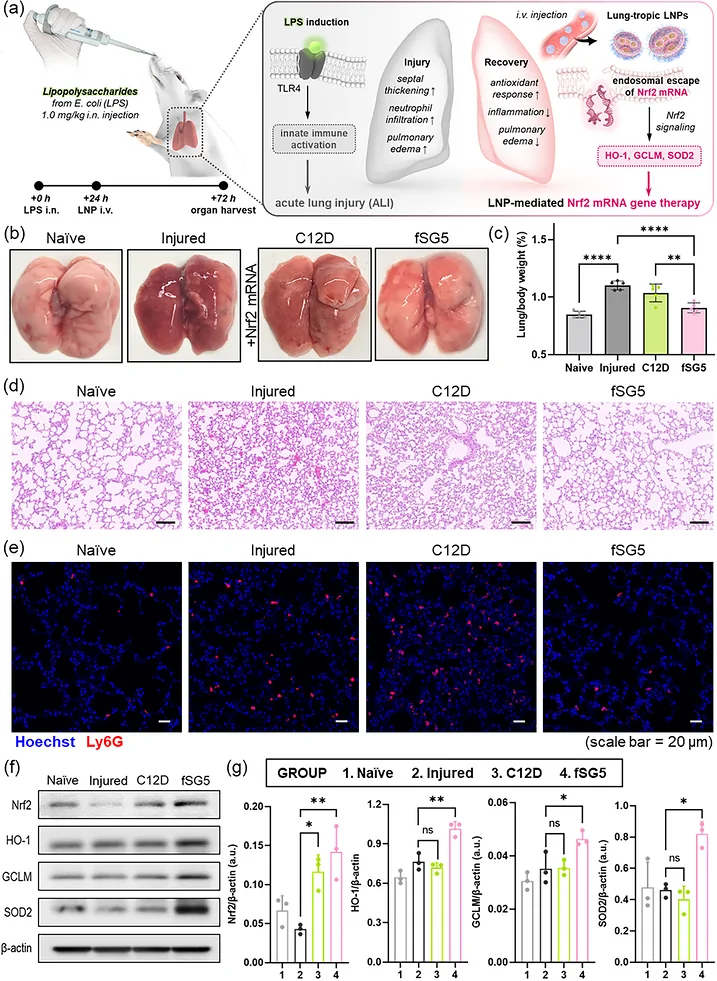
Therapeutic efficacy of lung‐tropic LNPs in an acute lung injury (ALI) model. (a) Schematic overview of ALI induction and i.v. gene therapy using lung‐tropic LNPs. (b) Representative gross images of lungs. (c) Lung edema index (*n = 5* biological replicates). (d) Representative images of the sliced lung tissues after H&E staining. Scale bar, 100 µm. (e) Representative immunohistochemistry (IHC for neutrophil) images of the sliced lung tissues. (f) Representative Western blot images for the indicated proteins from lungs. (g) Densitometric quantification of immunoblot bands normalized to β‐actin (*n = 3* biological replicates). LNPs were administered intravenously at 0.3 mg mRNA/kg for therapeutic efficacy studies. One‐way ANOVA with Tukey's multiple‐comparisons test, *p < 0.05, **p < 0.01, ***p < 0.0001, ****p < 0.00001.

Assessing the efficacy of gene delivery and expression in an injured environment is pivotal, as the goal of LNP development is gene‐based therapy rather than delivery alone. Therefore, we tested our lung‐tropic formulation in an acute lung injury (ALI) model using Nrf2 as a therapeutic gene [12]. In lipopolysaccharide (LPS)‐induced ALI, Toll‐like receptor 4 (TLR4) activation elicits a rapid innate immune response that elevates oxidative stress and pro‐inflammatory signaling, leading to pulmonary edema and tissue injury [71, 72]. This inflammatory state is associated with blunted induction of endogenous Nrf2 transcription [73], thereby limiting the activation of cytoprotective antioxidant pathways. To restore Nrf2 signaling under these conditions, we administered exogenous Nrf2 mRNA using lung‐tropic LNPs. In our previous work [74], Nrf2‐engineered hMSCs mitigated tissue injury, consistent with a protective role of Nrf2 signaling under oxidative stress. Building on this rationale, we hypothesized that Nrf2 mRNA delivery would confer therapeutic benefits in ALI, given the established role of Nrf2 in restraining oxidative stress and modulating inflammatory signaling [75, 76]. ALI was induced by intranasal administration of LPS from *Escherichia coli* (*E. coli*) O111:B4, followed 24 h later by a single intravenous injection of Nrf2 mRNA‐loaded LNPs (C12D or fSG5, 0.3 mg/kg mRNA dose). In treated animals, the lung‐tropic LNPs promoted Nrf2 pathway activation in the lung, as evidenced by the induction of downstream effectors, including HO‐1, GCLM, and SOD2 (Figure 7a).

Gross examination of the lungs at the endpoint revealed group‐dependent differences (Figure 7b). The naïve (uninjured) group exhibited a normal macroscopic appearance, whereas the injured lungs were diffusely congested. Both LNP‐treated groups showed an improved gross appearance compared with the injured group, with fSG5 more closely resembling naïve than C12D. These observations were consistent with the lung‐to‐body‐weight ratio (edema index) [77, 78], which increased from a median of 0.70% in naïve to 1.20% in injured mice and was 1.05% with C12D and 0.80% with fSG5 (Figure 7c). Accordingly, fSG5 more effectively mitigated LPS‐induced lung injury and edema than C12D, bringing both the gross morphology and edema index closer to naïve levels.

Hematoxylin and eosin staining confirmed the macroscopic findings (Figure 7d). Injured lungs exhibited alveolar septal thickening, interstitial edema, and inflammatory cell infiltration. LNP‐treated lungs displayed attenuation of these features, with C12D‐treated lungs showing partial improvement but retained residual inflammation and perivascular congestion. In the fSG5 group, lung sections showed features consistent with recovery, including reduced inflammatory burden and macrophage‐rich regions suggestive of the clearance of inflammatory lesions. To examine the degree of inflammation, we collected signals from neutrophils by immunoblotting with Ly6G antibody in sliced lung tissues (Figure 7e) [79]. To avoid signals from autofluorescence, Alexa 647‐conjugated secondary antibodies were used, with counterstaining of nuclei. In the injured sample, with an increased number of nuclei due to septal thickening, bright Ly6G‐labelled cells were increased compared to the naïve group, indicating the manifestation of inflammation. When treated with Nrf2 mRNA delivered by fSG5, the population of neutrophils sharply decreased and was similar to the level of naïve group, indicating the high effect of the gene therapy. In C12D group, retained residual inflammation was observed, consistent with the H&E results.

To examine the underlying mechanism, we performed immunoblotting for the Nrf2 pathway (Figure 7f,g). First, the level of Nrf2 was blotted with lungs after 6 h of LNP administration, which is 30 h post LPS induction. Consistent with prior reports, Nrf2 levels were reduced in the ALI model relative to naïve [80, 81] and were increased by both C12D and fSG5, with fSG5 exceeding C12D. The downstream effectors of Nrf2 [82] were also blotted after completion of the therapy. HO‐1, GCLM, and SOD2 were upregulated compared with the injured group, with the strongest induction observed in fSG5. Together, these data indicate that fSG5 successfully activated Nrf2‐dependent antioxidant responses, lowered cytokine burden, and reduced edema and histological injury in LPS‐induced ALI.

## 3 Conclusions

We demonstrated that an ionizable lipid and its formulated LNP exhibiting dual transition apparent pKa behavior can drive strong, lung‐selective mRNA expression, and that this performance extends to a disease‐relevant setting. Although we focused on a single chemistry series (SG2‐SG6) and one optimized formulation (fSG5), our findings motivate a broader structure‐function exploration of dual pKa motifs for pH‐programmed organ targeting and endosomal escape. In fSG5, two separable apparent pKa transitions enable stepwise protonation along the physiological‐to‐endocytic pH gradient, allowing access to distinct charge states in circulation and during intracellular trafficking.

We specifically propose that this stepwise charge‐state switching broadens the pH window over which the ionizable lipid engages with endosomal membranes and promotes cargo release. This provides a design rationale for chemistries that remain near‐neutral at pH 7.4, yet access higher cationic states as endosomes mature. Consistent with this model, molecular dynamics simulations have revealed protonation state‐dependent interaction modes at the membrane interface, suggesting that distinct charge states can favor different membrane engagement patterns. Beyond an expanded buffering range, this diversity of membrane interaction modes may contribute to the higher potency of the SG5‐based LNP.

Because organ selectivity, cytosolic delivery, and tolerability are often co‐governed by overlapping formulation variables, optimizing one objective can constrain the others. In this context, evaluation in both healthy and LPS‐injured mice provides a practical assessment of whether delivery performance is retained under therapy‐relevant tolerability constraints. In our case, fSG5 achieved high expression levels with a favorable tolerability profile. In the ALI model, the therapeutic benefit was consistent with the delivery advantage observed in healthy models. Although absolute equivalence across models was not assumed, the consistent ranking across carriers suggests that potency can be translated when tolerability liabilities are controlled. Future studies should test more severe or chronic models and broader dose ranges to delineate the limits of translatable therapeutic expression under tolerability constraints.

Several questions still remain. Despite the high DOTAP content of the comparator, fSG5 showed stronger lung expression than C12D. Protein corona composition and surface chemistry are potential contributors and motivate systematic studies using proteomics and surface characterization. Regarding tolerability, SG5‐based LNPs were well tolerated with lung selectivity even though they are mildly cationic near pH 7.4. The contrast with C12D may reflect the headgroup chemistry: SG5 contains titratable tertiary amines, whereas DOTAP provides a permanently charged quaternary ammonium. The generalizability of this tolerability across doses, species, and disease contexts remains to be established. Studies on clearance, metabolism, tissue residence, and dose‐response are therefore priorities. In parallel, more extensive simulations, including coarse‐grained approaches such as MARTINI, may help clarify how protonation‐dependent interactions translate into efficient endosomal escape while maintaining tolerability.

In summary, our findings outline a path that jointly advances lung‐targeting and efficient cytosolic release, with therapeutic relevance to acute inflammatory lung indications. Given the central role of oxidative stress in pulmonary injury, including influenza and SARS‐CoV‐2, the Nrf2‐axis and other cytoprotective mRNAs warrant further evaluation. For genetic indications such as surfactant protein deficiency or CFTR correction, lung‐tropic delivery paired with editing or repair cargo could also be assessed for local efficacy.

## 4 Experimental Section

### 4.1 Materials

C12‐200 (Cayman Chemical, Ann Arbor, MI, USA) and synthesized SG‐series lipids were used as ionizable lipids. Five epoxides were purchased from Tokyo Chemical Industry (Tokyo, Japan). DSPC, DOPC, DOPE, POPE, DOTAP, and C14‐PEG2000 were purchased from Avanti Polar Lipids (Alabaster, AL, USA). CleanCap reagent AG and modified/unmodified ribonucleotides were from TriLink BioTechnologies (San Diego, CA, USA). Cy5‐UTP was obtained from APExBIO (Houston, TX, USA). Unless otherwise stated, analytical‐grade reagents were from Sigma‐Aldrich (St. Louis, MO, USA). Reporter (luciferase) and Nrf2 mRNAs were generated in‐house by T7 polymerase in vitro transcription kit (TriLink) and purified (Monarch RNA Cleanup, NEB) as described previously [83]. Cre mRNA was purchased from GenScript (Piscataway, NJ, USA).

### 4.2 Chemical Synthesis of SG‐Series Ionizable Lipids

All reagents were used as received unless noted. SG‐series ionizable lipids were prepared by microwave‐assisted ring opening of 1,2‐epoxyalkanes with 1,2‐bis(methylamino)ethane. The SG5 analogues, isoSG5 and proSG5, were synthesized using the same synthetic procedure as SG5, with 3‐methylbutylamine and 3‐(dimethylamino)‐1‐propylamine used as the respective amine reagents. In a sealed microwave vial, 1,2‐bis(methylamino)ethane (limiting; 1.0 eq.) was combined with the corresponding 1,2‐epoxyalkane (epoxide in excess; typically 3–5 eq.; used neat as the reaction medium). The mixture was heated at 150 °C for 6 h under microwave irradiation (Initiator+, Biotage AB, Uppsala, Sweden) and then cooled to room temperature. Crude products were purified by flash chromatography on silica gel (CH_2_Cl_2_/MeOH gradients) using a Biotage Isolera MPLC system (Biotage AB, Uppsala, Sweden). Reaction progress was monitored by TLC (silica gel 60 F254, 0.25 mm) with visualization by ninhydrin or phosphomolybdic acid (PMA) followed by heating. A representative scale for the C14 homolog is provided (1,2‐epoxytetradecane 3.0 mL; 1,2‐bis(methylamino)ethane 370 µL, 3.4 mmol). Full schemes, exact scales and yields for each homolog, and characterization data (HRMS, 1H NMR; JEOL 500 MHz, δ in ppm vs TMS, *J* in Hz) are provided in the Supplementary Information.

### 4.3 Fabrication of mRNA‐LNPs

LNPs were formulated by rapid mixing of the ethanol and citrate buffer (20 mM, pH 3.0) phases at an aqueous:ethanol volume ratio of 3:1 using a Vortex‐Genie 2 (Scientific Industries) at maximum speed. Unless otherwise stated, the total lipid:mRNA weight ratio was 40:1 for C12‐200‐based LNPs and 80:1 for SG‐series LNPs. Crude LNPs were diluted with deionized water, concentrated using Amicon Ultra centrifugal filters (MWCO 10 kDa), and resuspended in DPBS (pH 7.4). All of the LNP concentrations in this paper is based on mRNA concentration unless expressed otherwise.

### 4.4 Statistical Analysis

Statistical analyses were performed using GraphPad Prism (v9.5, GraphPad Software). Data are presented as mean ± SD. For comparisons between two groups, an unpaired, two‐tailed Student's t‐test was used. For comparisons among three or more groups, one‐way ANOVA followed by Tukey's multiple comparisons test was used. *p < 0.05* was considered statistically significant. The definition of *n* and the number of biological replicates are provided in figure legends.

### 4.5 Physicochemical Characterization of LNPs

#### 4.5.1 Dynamic Light Scattering

LNP hydrodynamic diameter and zeta potential were measured using a Zetasizer Nano ZS (Malvern Panalytical, Malvern, UK) after a 30 s equilibration. Freshly prepared LNPs were diluted in deionized water to 0.5 ng/µL prior to measurement.

#### 4.5.2 Zeta Potential Titration

Apparent pKa values of LNPs were determined by zeta potential measurements across a pH range of 4–9. LNPs were diluted to 5 ng/µL in standard buffers and analyzed at 80 V using a Zetasizer Nano ZS (Malvern Panalytical, Malvern, UK) [49]. Each sample was measured in triplicate with 30 s equilibration. Nonlinear regression analysis was performed using a four‐parameter logistic model for single inflection curves and a five‐parameter logistic model for dual inflection curves.

#### 4.5.3 TNS Assay

Apparent pKa values were confirmed using a TNS (2‐(p‐toluidino)naphthalene‐6‐sulfonic acid) assay (Cayman Chemical, Ann Arbor, MI, USA) over a pH range of 4.0‐8.5 following the manufacturer's protocol.

#### 4.5.4 Laurdan Membrane Fluidity Assay

Laurdan dye was dissolved in DMSO to generate a 1 mM stock solution and stored at −20°C. LNPs were diluted in PBS (pH 7.4 or 5.5) to 0.5 mM total lipid concentration. Laurdan was added at a 1:250 molar ratio (final DMSO, 0.2% (v/v)). Samples were incubated at 37°C for 30 min. Fluorescence spectra were acquired using a Synergy H1 reader (Agilent, Santa Clara, CA, USA) with excitation at 340 nm and emission from 400 to 550 nm (10 nm intervals). Intensities were normalized, and generalized polarization (GP) values were calculated as described in Eq. 1.

(1) $$\mathrm{GP}=\frac{I_{420-450}-I_{470-520}}{I_{420-450}+I_{470-520}}\times 100\left(\%\right)$$

#### 4.5.5 Hemolysis Assay

LNPs (50 ng/µL) were diluted 1:1 with PBS at pH 7.4 or 5.5. Fresh mouse blood was diluted 9:1 with the same buffer to yield a 10% RBC suspension. Equal volumes of diluted LNP solution and RBC suspension were mixed and incubated at 37°C for 1 h. Mixtures were centrifuged (800 × g, 20 s), and 100 µL of supernatant was transferred to a 96‐well plate. PBS served as a negative control, and 0.5% Triton X‐100 in 10% RBC suspension was the positive control. Absorbance at 515 nm was recorded, and hemolysis (%) was calculated as described in Eq. 2.

(2) $$\mathrm{hemolysis}=\frac{I-\;I_{min}}{I_{max}}\times 100\left(\%\right)$$

### 4.6 Computational Analyses

#### 4.6.1 Molecular Docking Predictions

The structures of the ionizable and helper lipids were drawn in ChemDraw (PerkinElmer Inc., Waltham, MA, USA) and energy‐minimized using the MMFF94 force field. Docking predictions were performed using AutoDock Vina [84]. Docking input files (PDBQT) were prepared using AutoDockTools, in which polar hydrogens were added and Gasteiger partial charges were assigned. The grid box was defined to encompass the entire lipids. Docking poses were inspected, and representative poses showing hydrogen bonds were selected and visualization was conducted with The PyMOL Molecular Graphics System (Schrödinger, LLC., New York, NY, USA).

#### 4.6.2 Molecular Dynamics Simulations of Ionizable Lipid Endosome Insertion

All‐atom molecular dynamics simulations were performed using GROMACS (version 2025.3) [85, 86] on GPU workstations equipped with NVIDIA RTX 3060 or A6000 GPUs. Initial topology files and the membrane simulation systems were generated by CHARMM‐GUI [87, 88, 89]. Ionizable lipids were initially placed ∼6.5 nm away from the membrane center. To mimic a late endosomal membrane, the bilayer was composed of BMP, DOPE, and DOPC at a molar ratio of 5:3:2, with 80 lipids per leaflet. Energy minimization and six‐step sequential equilibration were performed according to the default protocol [87]. The initial simulation box size of 6.5 × 6.5 × 14 nm^3^ was relaxed to 7.183 × 7.183 × 11.083 nm^3^ during equilibration, corresponding to an area per lipid of approximately 0.65 nm^2^. The system was neutralized and ionized with 0.15 M NaCl and TIP3P water was used. Short‐range cutoffs for van der Waals and Coulomb interactions were set to 1.2 nm, and long‐range electrostatics were treated using the particle mesh Ewald (PME) method [90]. Hydrogen bonds were constrained using the LINCS algorithm [91]. During production, temperature (303.15 K) and pressure (1 bar) were controlled using the V‐rescale thermostat and the C‐rescale barostat under semi‐isotropic coupling [92]. A 2 fs time step was used, and simulations were run for a total of 600 ns, where ionizable lipid insertion events occurred within the first 100 ns and analysis was performed on the final 500 ns after insertion [63]. Five independent runs were conducted for each ionizable lipid species (*n = 5* per lipid; total *n = 10*). Trajectories were visualized using The PyMOL Molecular Graphics System (Schrödinger, LLC., New York, NY, USA).

#### 4.6.3 Molecular Dynamics Simulations and Trajectory Analysis of LNP Bilayer Membranes

Bilayer systems were constructed using the MolCube Membrane Builder, a commercially available platform analogous to the CHARMM‐GUI Membrane Builder [93]. Each leaflet contained 100 lipid molecules composed of 50 SG‐series ionizable lipids, 10 DOPE molecules, and 40 cholesterol molecules. The systems were solvated with TIP3P water and neutralized with 0.15 M NaCl. Following energy minimization and equilibration, production molecular dynamics simulations were performed for 500 ns.

Trajectories were analyzed using custom Python scripts based on MDAnalysis, NumPy, and SciPy. For tail‐splay analysis, a representative vector was defined for each hydrocarbon tail from the center of mass of the first two hydrophobic carbon atoms to that of the terminal three carbon atoms, and the angle between the two tail vectors was calculated for each ionizable lipid. Local lipid areas were estimated using periodic two‐dimensional Voronoi tessellation, with each lipid represented by a headgroup position projected onto the *xy* plane. The global APL was calculated by dividing the instantaneous lateral box area by the number of lipid molecules per leaflet. CPP_Rg_ was calculated as the ratio of the mass‐weighted lateral radius of gyration of the tail region to that of the headgroup region for each ionizable lipid. All geometric parameters were calculated for individual lipids in each analyzed frame and subsequently averaged across lipids and trajectory frames.

#### 4.6.4 Density Functional Theory (DFT) Calculations

Mono‐protonated molecular models of SG5 and proSG5 were prepared using Avogadro, with the proton added to the sterically less hindered amine nitrogen. Each structure was geometry optimized using GFN2‐xTB [94] with an implicit aqueous solvation model [95]. The optimized geometries were then used for single‐point DFT calculations in Psi4 [96] at the ωB97X‐D/def2‐SVP level [97, 98]. The total molecular charge was assigned as +1, and all calculations were performed as closed‐shell singlet states. Molecular electrostatic potential surfaces were generated by mapping DFT‐calculated electrostatic potentials onto electron density isosurfaces using Jmol. The electron density isosurface cutoff was fixed at 0.001 a.u., and the ESP color scale was fixed from −0.05 to +0.15 a.u. for all structures. Löwdin and Mulliken population analyses were used to estimate protonation‐induced C‐H polarization along the diamine bridge.

### 4.7 Cell Line

HeLa cells (RRID:CVCL_0030) were obtained from ATCC (Manassas, VA, USA) and cultured in RPMI‐1640 supplemented with 10% fetal bovine serum (Biowest USA, Inc., Bradenton, FL, USA) and 1% antibiotic‐antimycotic (Gibco, Thermo Fisher Scientific, Waltham, MA, USA) at 37°C in 5% CO_2_. Experiments were performed within ≤20 passages after thawing.

### 4.8 In Vitro Assays

#### 4.8.1 In Vitro Luciferase Assay

HeLa cells (1 × 10^4^ cells per well) were seeded in 96‐well clear‐bottom plates (Corning Incorporated, Corning, NY, USA) and incubated for 24 h. LNPs were added per well at 0.5 ng/µL mRNA dose (50 ng mRNA in 100 µL medium) and incubated overnight. Cells were washed twice with DPBS and lysed with 50 µL 1× passive lysis buffer (Promega Corporation, Madison, WI, USA; diluted from 5× stock). After 30 min incubation at room temperature with gentle shaking, 100 µL luciferin detection solution (freshly prepared at 0.6 mg/mL luciferin, 10 mM MgSO_4_, 2 mM coenzyme A, 1 mM ATP, and 40 mM Tris‐HCl, pH 7.8) was added per well. Luminescence was measured immediately using a Synergy H1 microplate reader (Agilent, Santa Clara, CA, USA; gain = 200).

#### 4.8.2 Endosomal Trafficking Analysis

HeLa cells (1 × 10^5^ per well) were seeded on sterilized round glass coverslips in 12‐well plates and cultured for 24 h. Cells were treated with LNPs (0.5 ng/ µL mRNA dose, 500 ng mRNA in 1 mL medium) for 1 h, washed with DPBS, and fixed with 4% PFA. Cells were permeabilized with 0.1% Triton X‐100 and incubated overnight with primary antibodies against EEA1 (Cat. #MA5‐14794, Thermo Fisher Scientific, Waltham, MA, USA; 1:200 in 0.1% BSA/DPBS) or LAMP1 (Cat. #SC‐20011, Santa Cruz Biotechnology, Inc, Dallas, TX, USA; 1:50 in 0.1% BSA/DPBS). After washing, cells were incubated for 1 h with Alexa Fluor 488‐conjugated anti‐rabbit secondary antibody and Alexa Fluor 555‐phalloidin. Nuclei were counterstained with Hoechst 33342, and coverslips were mounted using an aqueous mounting medium. Colocalization was quantified by Pearson's correlation coefficient using Fiji (ImageJ) with the Coloc 2 plugin. Two or three cells were randomly selected from each image, and a total of 10 cells per group were analyzed.

#### 4.8.3 Crystal Violet Staining

HeLa cells (1 × 10^5^ per well) were seeded in 12‐well plates and cultured for 24 h. LNPs were added according to the indicated dose (ng mRNA per well). After 24 h, cells were washed with DPBS, fixed with 30% ethanol/4% formaldehyde for 5 min, washed three times, and stained with 0.2% crystal violet (in methanol) for 10 min. Excess dye was removed by washing three times with distilled water, and plates were air‐dried. Images were obtained using an EVOS imaging system (Thermo Fisher Scientific, Waltham, MA, USA). For quantification, bound dye was eluted with 200 µL 1% SDS, diluted 1:10 in distilled water, and transferred to a 96‐well plate. Absorbance was measured, and values were normalized to untreated controls (100%).

#### 4.8.4 Cytotoxicity Assay (CCK‐8)

HeLa cells (1 × 10^4^ per well) were seeded in 96‐well plates and incubated for 24 h. Cells were treated with LNPs for 24 h, washed with DPBS, and incubated with CCK‐8 reagent (Dojindo Laboratories, Kumamoto, Japan) diluted 1:10 in HBSS (Biowest USA, Inc., Bradenton, FL, USA). After 1 h at 37°C in 5% CO_2_, absorbance at 450 nm was measured using a Synergy H1 reader (Agilent, Santa Clara, CA, USA).

### 4.9 Animal Experiments

All animal procedures were approved by the Institutional Animal Care and Use Committee (IACUC) of The Catholic University of Korea (approval no. 2025‐0098‐01) and were conducted in accordance with the Laboratory Animal Act (Republic of Korea), the Guide for the Care and Use of Laboratory Animals, and institutional standard operating procedures. Experiments complied with the ARRIVE guidelines. Animals were housed in a specific‐pathogen‐free (SPF) barrier facility with a 12 h light/dark cycle and ad libitum access to standard chow and water.

#### 4.9.1 Miles Assay

Evans blue was dissolved in DPBS to 0.5% (w/v), sterile‐filtered (0.2 µm, Minisart NML SFCA, Sartorius, Göttingen, Germany), aliquoted in amber tubes, and stored at −20°C. To measure in vivo toxicity of LNPs in the lung, healthy CD‐1 mice were intravenously injected with a high LNP dose (1.0 mg/kg mRNA dose). After 24 h, mice were injected with 100 µL/20 g of Evans blue via the tail vein and sacrificed 2 h later under isoflurane anesthesia. Lungs were perfused with ice‐cold DPBS through the right ventricle, excised, blotted dry, and stored at −80°C. For dye extraction, 20 µL formamide was added per 10 mg lung tissue and incubated at 55°C overnight. Supernatants were clarified by centrifugation (12 000 ×g, 15 min, 4°C) and 100 µL aliquots were transferred to 96‐well plates for absorbance measurement. Absorbance was measured at 620 nm with 740 nm as the reference, and corrected using Eq. 3. Dye content was calculated from a standard curve and expressed as µg dye per g of wet lung tissue.

(3) $$A_{620}\;\left(corrected\right)=A_{\;620}\;-\left(1.426\times A_{740}+0.030\right)$$

#### 4.9.2 Blood Chemistry

For blood chemistry analysis in healthy CD‐1 mice, animals were intravenously injected with a high LNP dose (1.0 mg/kg mRNA dose). After 24 h, mice were sacrificed and blood was collected via cardiac puncture. Whole blood was allowed to clot for 30 min at room temperature and centrifuged at 2000 × *g* for 20 min to obtain serum. Serum samples were stored at −80°C until analysis. Alanine aminotransferase (ALT) and aspartate aminotransferase (AST) levels were measured using a dry chemistry analyzer (DRI‐CHEM NX500, Fujifilm, Tokyo, Japan).

#### 4.9.3 Hematoxylin and Eosin (H&E) Staining

Organs from the same CD‐1 mice used for blood chemistry were harvested, gently washed in DPBS, and fixed in 4% paraformaldehyde solution (PFA; Dreamcell, Korea) for 24 h at room temperature. Fixed tissues were dehydrated through graded ethanol, embedded in paraffin blocks, and sectioned at 10 µm thickness. Sections were stained with hematoxylin and eosin (H&E) following a standard protocol and imaged using an EVOS imaging system (Thermo Fisher Scientific, USA).

#### 4.9.4 Lung Injury Animal Model

Male CD‐1 mice (5–6 weeks old; approximately 30 g; Oriental Bio, Korea) were used. Lipopolysaccharide (LPS; *E. coli* O111:B4, Sigma‐Aldrich, Cat. #L4391) was prepared at 2 mg/mL in DPBS, sterile‐filtered (0.2 µm, Sartorius, Göttingen, Germany), aliquoted, and stored at −20°C; each aliquot was thawed once immediately before use. For all procedures, mice were anesthetized in an isoflurane induction chamber (induction 2.5‐3.0% for 5–10 min; maintenance 2.0% by brief re‐exposure to the chamber as needed). Depth of anesthesia was confirmed by loss of pedal withdrawal reflex. Mice received intranasal instillation of LPS at 1.0 mg/kg in a weight‐normalized total volume of 0.5 µL/g (e.g., 15 µL for a 30 g mouse), administered dropwise into the external nares as alternating small drops between sides while the animal was breathing spontaneously. At 24 h after LPS instillation, mice were randomly assigned to receive a single intravenous (tail vein) dose of Nrf2 mRNA‐loaded LNPs, either C12D or fSG5, at 0.30 mg/kg mRNA dose. Experimental groups were: Naïve (uninjured), LPS only (acute lung injury), LPS + C12D, and LPS + fSG5 (*n = 5* per group). At 72 h after LPS, mice were terminally anesthetized with isoflurane; blood was collected by cardiac puncture under deep anesthesia, and cervical dislocation was performed to ensure death. Lungs were excised without perfusion and gently rinsed with ice‐cold DPBS before downstream analysis.

#### 4.9.5 Immunohistochemistry on Paraffin‐Embedded Tissues (IHC‐P)

Lung tissues harvested at the end of the therapeutic study were fixed, paraffin‐embedded, and sectioned (10 µm). Sections were baked at 60°C for 60 min, cooled to room temperature, deparaffinized in xylene (3×), and rehydrated through graded ethanol to water. Heat‐induced epitope retrieval was performed in citrate buffer (pH 6.0; Cat. #ZUC028‐500, ZytoVision GmbH, Bremerhaven, Germany) using a Coplin jar immersed in a 92°C water bath for 30 min, followed by cooling at room temperature for 20 min and rinsing in tap water and then DPBS. Sections were permeabilized with 0.1% Triton X‐100 in DPBS, blocked in 3% BSA with 0.1% Tween 20 in DPBS, and incubated overnight at 4°C with anti‐Ly6G (Cat. #87048, Cell Signaling Technology, Danvers, MA, USA; 1:200 in 0.5% BSA/DPBS). After three washes in 0.05% Tween 20/DPBS, slides were incubated for 1 h at room temperature with Alexa Fluor 647‐conjugated goat anti‐rabbit secondary antibody (1:200 in 0.5% BSA/DPBS). Slides were washed in DPBS and treated with TrueBlack (Cat. #23007, Biotium, Fremont, CA, USA) for 1 min to reduce lung autofluorescence. Finally, nuclei were counterstained with Hoechst 33342 for 3 min and mounted with an aqueous medium. Fluorescence images were acquired on a confocal laser‐scanning microscope (FV3000, Evident Corporation, Tokyo, Japan); acquisition settings were kept constant within each experiment.

#### 4.9.6 Lung Tissue Protein Extraction and Western Blot

Harvested lungs were gently washed with DPBS and wet lung weights were recorded. Lungs for Nrf2 immunoblotting were harvested 6 h after LNP injection, whereas lungs for immunoblotting of Nrf2 downstream targets (HO‐1, GCLM, and SOD2) were harvested at the end of the therapeutic study. For protein extraction from tissues, RIPA buffer supplemented with protease inhibitor was applied at a ratio of 10 µL per 20 mg of wet tissue. Homogenization was performed using a handheld tissue grinder motor, and the lysates were centrifuged at 13 000 × *g* for 15 min to collect the supernatant. Total protein concentrations were determined using a BCA protein assay kit (Thermo Fisher Scientific, Waltham, MA, USA). For SDS‐PAGE, 25 µg of total protein was mixed with 4× Laemmli sample buffer (Bio‐Rad, Hercules, CA, USA) to a final volume of 20 µL and boiled at 95°C for 10 min. Proteins were separated and transferred onto PVDF membranes (Immune‐blot, Cat. #1620177, Bio‐Rad, Hercules, CA, USA), and probed with antibodies against Nrf2 (Cat. #ab137550, Abcam, Cambridge, UK), HO‐1 (Cat. #ab13248, Abcam, Cambridge, UK), GCLM (Cat. #33381, Cell Signaling Technology, Danvers, MA, USA), and SOD2 (Cat. #13194, Cell Signaling Technology, Danvers, MA, USA). β‐actin antibody (Cat. #8457, Cell Signaling Technology, Danvers, MA, USA) was used as a loading control. All experiments were performed with *n = 3* biological replicates (mice) per group. Uncropped immunoblot membrane images are provided in Figure S22.

#### 4.9.7 Serum ELISA Analysis

For serum ELISA analysis following repeated administration of fSG5, mice received a single or repeated intravenous injection of fSG5 at an mRNA dose of 0.30 mg/kg. Serum was collected by cardiac puncture at 6 or 24 h after the final administration. Whole blood was allowed at room temperature for 30 min and then centrifuged at 2000 × *g* for 20 min to isolate the serum. Serum samples were stored at −80°C until analysis. Serum concentrations of TNF‐α and IL‐6 were measured using commercially available ELISA kits from KOMA Biotech (TNF‐α, Cat. #K0331186;IL‐6, Cat. #K0331230) according to the manufacturer's instructions.

### 4.10 In Vivo Imaging

#### 4.10.1 In Vivo Luciferase Imaging

Male CD‐1 mice (6–8 weeks old, RRID:IMSR_CRL:022) were used for in vivo transfection experiments. LNPs were administered via tail vein at the 0.25 mg/kg mRNA dose. After 6 h, mice were anesthetized with isoflurane, and injected intraperitoneally with 100 mg/kg D‐luciferin (Promega Corporation, Madison, WI, USA). Whole‐body luminescence images were acquired 7 min post‐injection using an IVIS Spectrum (PerkinElmer Inc., Waltham, MA, USA). Ex vivo organ imaging was performed in the same animals after sacrifice.

#### 4.10.2 Ex Vivo tdTomato Fluorescence

ROSA26 reporter mice (R26R‐tdTomato floxed; RRID:IMSR_JAX:003474) were obtained from the Laboratory Animal Resource Center (LARRC), Korea Research Institute of Bioscience and Biotechnology (KRIBB). Cre mRNA‐loaded LNPs (0.25 mg/kg mRNA dose) were intravenously injected into mice. To allow sufficient Cre‐mediated recombination and tdTomato expression, mice were euthanized 72 h post‐injection. Ex vivo fluorescence of excised organs was assessed with IVIS and quantified using Living Image software. Tissues were fixed in 4% paraformaldehyde overnight at 4°C, cryoprotected in sucrose (10%, 15%, and 20% in DPBS), embedded in OCT, snap‐frozen, and stored at −80°C. Cryosections (10 µm thickness) were prepared within 2 weeks of harvest using a cryostat (CM1850, Leica, Germany). Sections were mounted on coated glass slides, counterstained with Hoechst 33342, and imaged using an Eclipse Ti2 fluorescence microscope (Nikon Instruments, Melville, NY, USA).

## Conflicts of Interest

The authors declare no conflict of interest

## Supporting information




**Supporting File**: advs77387‐sup‐0001‐SuppMat.docx.
