# Supplementary material for: Dual pKa Lipid Nanoparticles for Lung‐tropic mRNA Delivery and pH‐Programmed Endosomal Escape
Source: Adv Sci (Weinh). 2026 Sep 9:e77387. Online ahead of print. doi: 10.1002/advs.77387 (PMC13556983; doi:10.1002/advs.77387)
Supplement: Supplementary file 1 [file ADVS-9999-e77387-s001.docx]

Supporting Information

Dual pKa lipid nanoparticles for lung-tropic mRNA delivery and pH-programmed endosomal escape

Seong Gi Lim, Minju Lee, Yiming Wang, Eunjin Choi, Ji Sun Park, Kyungmin Park, Hayoung Jeon, Donghyun Kim, Donghyun Lee, Yeeun Lee, Kee-Pyo Kim, Eunha Kim, and Heebeom Koo^*^

**Methods**

**Synthesis of SG-series ionizable lipids**

Splitting patterns are d = doublet, t = triplet, m = multiplet. Chemical shifts (δ) are reported in ppm with reference to tetramethylsilane (TMS) as an internal standard. Coupling constant (*J*) is given in Hz.

**SG2**	1,2-Bis(methylamino)ethane (500 μL, 4.58 mmol) was added to a solution of 1,2-epoxydecane (3.4 mL, 18.32 mmol). The reaction mixture was stirred for 6 h at 150 °C in microwave reactor. The mixture was purified by flash column chromatography (MeOH/MC) to give a pale-yellow oil (403.8 mg, 22.0%). ^1^H NMR (500 MHz, CDCl_3_) δ 3.65 – 3.49 (m, 2H), 2.80 – 2.25 (m, 7H), 2.25 – 2.22 (m, 6H), 2.17 – 2.09 (m, 1H), 1.46 – 1.23 (m, 28H), 0.88 – 0.84 (m, 6H) (**Fig. S1**). HRMS (ESI): [M+H]^+^ m/z calcd. 401.41016 for C_24_H_52_N_2_O_2_^+^, found 401.41031.

**SG3**	1,2-Bis(methylamino)ethane (500 μL, 4.58 mmol) was added to a solution of 1,2-epoxydodecane (4 mL, 18.32 mmol). The reaction mixture was stirred for 6 h at 150 °C in microwave reactor. The mixture was purified by flash column chromatography (MeOH/MC) to give a white solid (384.6 mg, 18.4%). ^1^H NMR (500 MHz, CDCl_3_) δ 3.65 – 3.50 (m, 2H), 2.79 – 2.28 (m, 7H), 2.24 (d, *J* = 8.8 Hz, 6H), 2.16 – 2.10 (m, 1H), 1.25 (d, *J* = 9.8 Hz, 36H), 0.88 – 0.85 (m, 6H) (**Fig. S2**). HRMS (ESI): [M+H]^+^ m/z calcd. 457.47276 for C_28_H_60_N_2_O_2_^+^, found 457.47293.

**SG4**	1,2-Bis(methylamino)ethane (500 μL, 4.58 mmol) was added to a solution of 1,2-epoxytetradecane (4.6 mL, 18.32 mmol). The reaction mixture was stirred for 6 h at 150 °C in microwave reactor. The mixture was purified by flash column chromatography (MeOH/MC) to give a colorless oil (566.6 mg, 24.1%). ^1^H NMR (500 MHz, CDCl_3_) δ 3.55 (m, 2H), 2.79 – 2.28 (m, 7H), 2.24 (d, *J* = 10.1 Hz, 6H), 2.13 (m, 1H), 1.41 – 1.23 (m, 44H), 0.87 (t, *J* = 6.9 Hz, 6H) (**Fig. S3**). HRMS (ESI): [M+H]^+^ m/z calcd. 513.53536 for C_32_H_68_N_2_O_2_^+^, found 513.53522.

**SG5**	1,2-Bis(methylamino)ethane (370 μL, 3.4 mmol) was added to a solution of 1,2-epoxyhexadecane (3.4 mL, 13.6 mmol). The reaction mixture was stirred for 6 h at 150 °C in microwave reactor. The mixture was purified by flash column chromatography (MeOH/MC) to give a pale-yellow oil (465.0 mg, 24%). ^1^H NMR (500 MHz, CDCl_3_) δ 3.55 (m, *J* = 17.1, 8.0, 4.7 Hz, 2H), 2.80 – 2.27 (m, 7H), 2.26 – 2.20 (m, 6H), 2.17 – 2.07 (m, 1H), 1.43 – 1.23 (m, 52H), 0.88 – 0.85 (m, 6H) (**Fig. S4**). HRMS (ESI): [M+H]^+^ m/z calcd. 569.59796 for C_36_H_76_N_2_O_2_^+^, found 569.59796.

**SG6**	1,2-Bis(methylamino)ethane (500 μL, 4.58 mmol) was added to a solution of 1,2-epoxyoctadecane (4.9 g, 15.25 mmol). The reaction mixture was stirred for 6 h at 150 °C in microwave reactor. The mixture was purified by flash column chromatography (MeOH/MC) to give a white powder (466.0 mg, 16.3%). ^1^H NMR (500 MHz, CDCl_3_) δ 3.63 – 3.49 (m, 2H), 2.79 – 2.26 (m, 7H), 2.26 – 2.21 (m, 6H), 2.15 – 2.11 (m, 1H), 1.24 (s, 60H), 0.87 (td, *J* = 7.0, 1.2 Hz, 6H) (**Fig. S5**). HRMS (ESI): [M+H]^+^ m/z calcd. 625.66056 for C_40_H_84_N_2_O_2_^+^, found 625.66040.

**isoSG5**	1-Amino-3-methylbutane (100 mg, 1.15 mmol) was added to a solution of 1,2-epoxyhexadecane (3.06 g, 11.5 mmol). The reaction mixture was stirred for 6 h at 150 °C in microwave reactor. The mixture was purified by flash column chromatography (MeOH/MC) to give a yellow oil (9.8 mg, 1.6 %). ^1^H NMR (500 MHz, CDCl_3_) δ 2.86 (tdd, *J* = 5.5, 4.0, 2.7 Hz, 2H), 2.70 (dd, *J* = 5.1, 4.0 Hz, 2H), 2.42 (dd, *J* = 5.1, 2.7 Hz, 2H), 1.48 – 1.15 (m, 60H), 0.91 – 0.81 (m, 9H). (**Fig. S6**). LCMS (ESI): [M+H] ^+^ m/z calcd. 569.7 for C_37_H_77_NO_2_^+^, found 569.7.

**proSG5**	3-(Dimethylamino)propylamine (1.26 mL, 10.0 mmol) was added to a solution of 1,2-epoxyhexadecane (1.20 g, 50.0 mmol). The reaction mixture was stirred for 12 h at 150 °C in microwave reactor. The mixture was purified by flash column chromatography (MeOH/MC) to give a white powder (409.8 mg, 7.0 %). ^1^H NMR (600 MHz, CD_3_OD) δ 3.61 – 3.53 (m, 2H), 3.47 (dd, *J* = 11.1, 4.5 Hz, 2H), 3.41 (dd, *J* = 11.1, 6.5 Hz, 2H), 1.59 – 1.17 (m, 62H), 0.90 (t, *J* = 7.0 Hz, 7H). (**Fig. S7**). LCMS (ESI): [M+H] ^+^ m/z calcd. 583.7 for C_37_H_78_N_2_O_2_^+^, found 583.7.

**Figure S1.** The NMR data and structure (inset)of SG2 lipid; ^1^H-NMR (CDCl_3_, 500MHz).

**Figure S2.** The NMR data and structure (inset) of SG3 lipid; ^1^H-NMR (CDCl_3_, 500MHz).

**Figure S3.** The NMR data and structure (inset) of SG4 lipid; ^1^H-NMR (CDCl_3_, 500MHz).

**Figure S4.** The NMR data and structure (inset) of SG5 lipid; ^1^H-NMR (CDCl_3_, 500MHz).

**Figure S5.** The NMR data and structure (inset) of SG6 lipid; ^1^H-NMR (CDCl_3_, 500MHz).

**Figure S6.** The NMR data and structure (inset) of isoSG5 lipid; ^1^H-NMR (CDCl_3_, 500MHz).

**Figure S7.** The NMR data and structure (inset) of proSG5 lipid; ^1^H-NMR (CD_3_OD, 600MHz).

**Figure S8.** Comparison of *ex vivo* luminescence intensity of mice 6 h after treated with LNPs of SG-series ionizable lipids at the 0.25 mg/kg mRNA dose. LNPs were fabricated in the conventional lipid ratio of 50:10:38.5:1.5 and total lipids were mixed with 1/40 of luciferase mRNA.

**Figure S9.** Global area per lipid (APL) distributions of SG2-SG6 bilayer models during the 500 ns of molecular dynamics simulations. Dashed lines indicated the median and quartiles.

**Figure S10.** Time-averaged CPP_Rg_ values of SG-series ionizable lipids with a linear regression fit.

**Figure S11.** *In vivo* comparison of SG5-based LNPs formulated using literature reported lipid ratios. All formulations were prepared at a total lipid to mRNA weight ratio of 40:1. Formulation ratios and corresponding references are provided in Table S1.

**Figure S12.** Relative luminescence of SG5-based LNPs in vitro formulated using established lipid ratios reported in the literature and a randomly selected ratio (Random), normalized to untreated cells. All formulations were prepared at a total lipid to mRNA weight ratio of 40:1. Formulation ratios and corresponding references are provided in Table S1.

**Figure S13.** *In vivo* comparison of SG5-based LNPs corresponding to the top three highest luminescence candidates identified from the second Taguchi array *in vitro*, for determination of the final SG5-based formulation (fSG5). All formulations were prepared at a total lipid to mRNA weight ratio of 80:1.

**Figure S14. *In vivo* comparison of fSG5 formulated at a total lipid:mRNA weight ratio of 40:1 (40:1 fSG5).** (a) Whole-body bioluminescence images of CD-1 mice intravenously administered 40:1 fSG5, displayed using the same radiance scale as in Fig. 2f. (b) *Ex vivo* luminescence images of major organs collected from the same mice (*n = 4* biological replicates), displayed using the same radiance scale as in Fig. 2e. (c) Quantification of average radiance in *ex vivo* lungs, plotted using the same y-axis scale as in Fig. 2g. (d) Average radiance in *ex vivo* lungs normalized to the mean value of the 40:1 fSG5 group (40:1 fSG5 = 1). The normalized values for C12D and optimized fSG5 were 0.2739 and 2.1276, respectively.

**Figure S15.** Physicochemical characterization of fSG5 and C12D LNPs. (a) Hydrodynamic diameter and polydispersity index (PDI). (b) Zeta potential.

**Figure S16.** Sirius T3 results of standard pKa titration of SG5 lipid.

**Figure S17.** Analyses of pKa values of C12D LNP via TNS assay and zeta-potential titration. Measurements were conducted three times for each point, and interpolation was conduction using sigmoidal, 4PL, log(concentration) model.

**Figure S18.** Mean Mulliken partial charges of bridge C-H hydrogens in mono-protonated SG5 and proSG5. Blue, green, and orange indicate proximal, middle, and distal positions, respectively.

**Figure S19.** Distal-to-proximal ratios of the mean Mulliken H partial charges of SG5 and proSG5.

**Figure S20.** Sirius T3 results of standard pKa titration of C12-200 lipid.

**Figure S21.** Toxicity evaluation of fSG5 following single and repeated administration.

(a) Experimental timelines for single and repeated administration. Serum concentrations of (b) TNF-α and (c) IL-6 measured by ELISA. Original Western blot membranes for (d) MPO, (e) iNOS, and (f) β-actin, with lanes arranged in the order of control, S6, S24, R6, and R24. (g) Densitometric quantification of MPO and iNOS expression normalized to β-actin.

**Figure S22.** Uncropped original immunoblot membrane images for (a) Nrf2 (~97 kDa) and β-actin (~42 kDa), and (b) downstream proteins HO-1 (~33 kDa), GCLM (~28 kDa), SOD2 (~24 kDa), and β-actin, presented in the group order naïve, injured, C12D, and fSG5. Samples were resolved on (a) 10% or (b) 12% Tris-glycine SDS-PAGE, as indicated. To enable direct comparison across targets, proteins were detected sequentially on the same membrane with stripping between probing steps. β-actin was probed on the corresponding membrane for normalization. Molecular weight markers and target positions were annotated for clarity; no other image processing was applied.

**Tables**

| **Reference paper** | | **SG5** | **DOPE** | **Chol** | **PEG** | **Ratio** |
| --- | --- | --- | --- | --- | --- | --- |
| Onpattro^[33]^ | | 18.3 | 3.7 | 14.1 | 0.5 | 50:10:38.5:1.5 |
| JACS 2012^[37]^ | | 24.1 | 2.5 | 9.2 | 0.3 | 66.6:7.0:25.5:0.9 |
| AM 2016^[35]^ | | 12.4 | 5.7 | 16.5 | 0.9 | 35:16:46.5:2.5 |
| SA 2021^[36]^ | | 9.8 | 7.4 | 19.2 | 0.6 | 26.5:20:52:1.5 |
| Random | | 32.1 | 0.5 | 2.9 | 0.2 | 90:1.5:8.0:0.5 |
| **1^st^**  **Taguchi** | **Name** | **24/28/32** | **0.5/2.5/5.5** | **3/6/9** | **0.1/0.2/0.3** | **Ratio** |
|  | 1T1 | 24 | 0.5 | 3 | 0.1 | 87:1.8:10.9:0.4 |
|  | 1T2 | 24 | 2.5 | 6 | 0.2 | 73.4:7.6:18.3:0.6 |
|  | 1T3 | 24 | 5.5 | 9 | 0.3 | 61.9:14.2:23.2:0.8 |
|  | 1T4 | 28 | 0.5 | 6 | 0.3 | 80.5:1.4:17.2:0.9 |
|  | 1T5 | 28 | 2.5 | 9 | 0.1 | 70.7:6.3:22.7:0.3 |
|  | 1T6 | 28 | 5.5 | 3 | 0.2 | 76.3:15.0:8.2:0.5 |
|  | 1T7 | 32 | 0.5 | 9 | 0.2 | 76.7:1.2:21.6:0.5 |
|  | 1T8 | 32 | 2.5 | 3 | 0.3 | 84.7:6.6:7.9:0.8 |
|  | 1T9 | 32 | 5.5 | 6 | 0.1 | 73.4:12.6:13.8:0.2 |
| **2^nd^**  **Taguchi** | **Name** | **30/32/34** | **0.2/0.5/1.5** | **7/9/11** | **0.15/0.20/0.25** | **Ratio** |
|  | 2T1 | 30 | 0.2 | 7 | 0.15 | 80.3:0.5:18.7:0.4 |
|  | 2T2 | 30 | 0.5 | 9 | 0.20 | 75.6:1.3:22.7:0.5 |
|  | 2T3 | 30 | 1.5 | 11 | 0.25 | 70.2:3.5:25.7:0.6 |
|  | 2T4 | 32 | 0.2 | 9 | 0.25 | 77.2:0.5:21.7:0.6 |
|  | 2T5 | 32 | 0.5 | 11 | 0.15 | 73.3:1.1:25.2:0.3 |
|  | 2T6 | 32 | 1.5 | 7 | 0.20 | 78.6:3.7:17.2:0.5 |
|  | 2T7 | 34 | 0.2 | 11 | 0.20 | 74.9:0.4:24.2:0.4 |
|  | 2T8 | 34 | 0.5 | 7 | 0.25 | 81.4:1.2:16.8:0.6 |
|  | 2T9 | 34 | 1.5 | 9 | 0.15 | 76.1:3.4:20.2:0.3 |

**Table S1.** Number of moles of LNP components used in this study. For reference formulations, mole numbers were calculated and normalized to the same mRNA mass to enable direct comparison across compositions. The literature formulation JACS 2012 exhibited the highest in vivo efficacy among the previously reported formulations (Fig. S7), and together with a randomly selected formulation (Random), showed the two highest in vitro luminescence values (Fig. S8). Accordingly, these two formulations were used to define the design space for the first Taguchi screening. The second Taguchi screening was centered around 1T7, corresponding to combination #7 in the first Taguchi design, and used a narrower mole-number range for finer optimization.
